# Comparison of switching bipolar ablation with multiple cooled wet electrodes and switching monopolar ablation with separable clustered electrode in treatment of small hepatocellular carcinoma: A randomized controlled trial

**DOI:** 10.1371/journal.pone.0192173

**Published:** 2018-02-08

**Authors:** Won Chang, Jeong Min Lee, Dong Ho Lee, Jeong Hee Yoon, Yoon Jun Kim, Jung Hwan Yoon, Joon Koo Han

**Affiliations:** 1 Department of Radiology, Seoul National University Bundang Hospital, 82, Gumi-ro 173 Beon-gil, Bundang-gu, Seongnam-si, Gyeonggi-do, Korea; 2 Department of Radiology, Seoul National University Hospital, Jongno-gu, Seoul, Korea; 3 Seoul National University College of Medicine, Jongno-gu, Seou, Korea; 4 Department of Internal Medicine, Seoul National University Hospital, Jongno-gu, Seoul, Korea; Yonsei University College of Medicine, REPUBLIC OF KOREA

## Abstract

**Objective:**

A randomized controlled trial was conducted to prospectively compare the therapeutic effectiveness of switching bipolar (SB) radiofrequency ablation (RFA) using cooled-wet electrodes and switching monopolar (SM) RFA using separable clustered (SC) electrodes in patients with hepatocellular carcinomas (HCCs).

**Materials and methods:**

This prospective study was approved by our Institutional Review Board. Between April 2014 and January 2015, sixty-nine patients with 74 HCCs were randomly treated with RFA using either internally cooled-wet (ICW) electrodes in SB mode (SB-RFA, n = 36) or SC electrodes in SM mode (SM-RFA, n = 38). Technical parameters including the number of ablations, ablation time, volume, energy delivery, and complications were evaluated. Thereafter, 1-year and 2-year local tumor progression (LTP) free survival rates were compared between the two groups using the Kaplan-Meier method.

**Results:**

In the SB-RFA group, less number of ablations were required (1.72±0.70 vs. 2.31±1.37, *P* = 0.039), the ablation time was shorter (10.9±3.9 vs.14.3±5.0 min, *p* = 0.004), and energy delivery was smaller (13.1±6.3 vs.23.4±12.8 kcal, *p*<0.001) compared to SM-RFA. Ablation volume was not significantly different between SB-RFA and SM-RFA groups (61.8±24.3 vs.54.9±23.7 cm^3^, *p* = 0.229). Technical failure occurred in one patient in the SM-RFA group, and major complications occurred in one patient in each group. The 1-year and 2-year LTP free survival rates were 93.9% and 84.3% in the SB-RFA group and 94.4% and 88.4% in the SM-RFA group (*p* = 0.687).

**Conclusion:**

Both SB-RFA using ICW electrodes and SM-RFA using SC electrodes provided comparable LTP free survival rates although SB-RFA required less ablations and shorter ablation time.

## Introduction

Radiofrequency ablation (RFA) is currently accepted as the treatment of choice for patients with very early stage or early stage hepatocellular carcinoma (HCC) when liver transplantation or surgical resection is not feasible [1–4]. According to a recent systematic review, RFA was shown to provide similar quality-adjusted life expectancy for very early HCCs (single nodule <2 cm) in Child–Pugh Class A patients at a lower cost compared with surgical resection [5]. However, although RFA provided comparable survival rates to surgical resection in patients with small HCCs (<3 cm), higher local tumor progression (LTP) rates have also been reported [6, 7]. Therefore, the creation of a sufficient ablative margin (>5 mm) around the target tumor is recommended in order to lower the LTP after RFA [8, 9]. Yet, in clinical practice, there is substantial technical difficulty in covering the entire tumor volume with a sufficient ablative margin using a single electrode, as it requires multiple overlapping ablations [10]. Under ultrasound guidance, repositioning the electrode for overlapping ablations carries technical complexity owing to gas bubble formation, thereby increasing procedure times and the potential of complications [11]. To overcome these limitations, multiple-electrode RFA approaches using switching monopolar, or multipolar RF energy delivery modes have been investigated, and several studies have demonstrated that they indeed provide better local therapeutic efficacy than the single electrode approach [12–15].

As of now, a monopolar RFA system is most commonly used for image-guided thermal ablation [10, 16]. In principle, with bipolar RFA, the electrical current flows between a pair of electrodes and a higher current density is maintained between electrodes [17, 18]. This electrophysiological feature of the bipolar RF delivery mode can allow rapid heating as well as less perfusion-mediated heat sink effect [10, 19, 20]. Recently, switching bipolar (SB) or multi-bipolar RFA using multiple electrodes was demonstrated to create large, regular ablation zones, potentially resulting in lower local tumor progression rates than switching monopolar (SM)-RFA in preclinical and clinical studies [20–24]. This may be attributable to the better heat production efficiency of bipolar RFA at any given current level compared to monopolar RFA[17]. However, despite of the potential benefit of SB- or multi-bipolar RFA in creating a larger ablation zone within a shorter ablation time all in a single session, there has not been any study performed prospectively in the clinical setting.

Thus, the purpose of this randomized clinical trial was to prospectively compare, in a random fashion, the therapeutic effectiveness and safety of SB-RFA versus those of SM-RFA in patients with HCCs.

## Materials and methods

This study received a research grant from RF Medical Co. (Seoul, Republic of Korea). All authors had complete control of all the data and information submitted for publication at all times.

### Study population

The institutional review board of Seoul National University Hospital approved this prospective, randomized single center study (#1310-051-526), and written informed consent was obtained from all patients. This study was conducted by Seoul National University Hospital, Korea and was additionally registered at ClinicalTrials.gov (NCT02675894). Although registration before patient enrollment was recommended, we did not register this study at the beginning, but to declare our study for the researcher, we registered our study on follow-up period. The authors confirm that all ongoing and related trials for this intervention are registered. As far as we know, this study was the first explorative study to therapeutic effectiveness and safety of SB-RFA and SM-RFA. The primary endpoint of the current study was the rate of LTP of the treated lesions, as there have been no studies of direct head to head comparison of two different RFA modes regarding LTP in a randomized clinical trial format. In our institution, approximately 150 patients with HCCs were treated by RFA annually. We assumed that among those patients, 70 patients could be enrolled in this study. And assuming the drop rate as 10%, size of the target population was determined as 77. Between April 2014 and January 2015, 77 patients who met our inclusion criteria below were enrolled in this study. We used the stratified permuted block randomization method on the size of largest HCC in each patient (small (1~2.5 cm) and medium (≥2.5 cm) HCC) with the fixed block size of 4. Patients were randomly assigned (1:1) into the SB-RFA group and SM-RFA group. Randomization process was performed using electrically generated random numbers and managed by our medical research collaboration center. Our inclusion criteria were as follows: (1) pathologic or typical imaging-based diagnosis of HCC; (2) dynamic CT or MRI scan within 60 days prior to RFA; (3) no more than three tumors, with with a diameter ranging from 1.0 cm to 5.0 cm; (4) Child–Pugh A or B liver function status; (5) no contraindication to conventional RFA such as uncooperative patients, intractable ascites, and uncorrectable coagulopathy bleeding; (6) absence of extrahepatic metastases on CT or MRI scan prior to RFA; (7) patients whose ages ranged from 20 to 75 years old; (8) no interventional treatment for hepatic index tumors such as transarterial chemoembolization, percutaneous RFA, or ethanol injection prior to RFA; and (9) patients who are going to undergo RFA for curative purposes. Patients were kept blinded to which group were allocated to, i.e. the SB-RFA group or SM-RFA group. In addition, outcome assessors were also kept blinded to the allocated treatment group.

**Fig 1:**
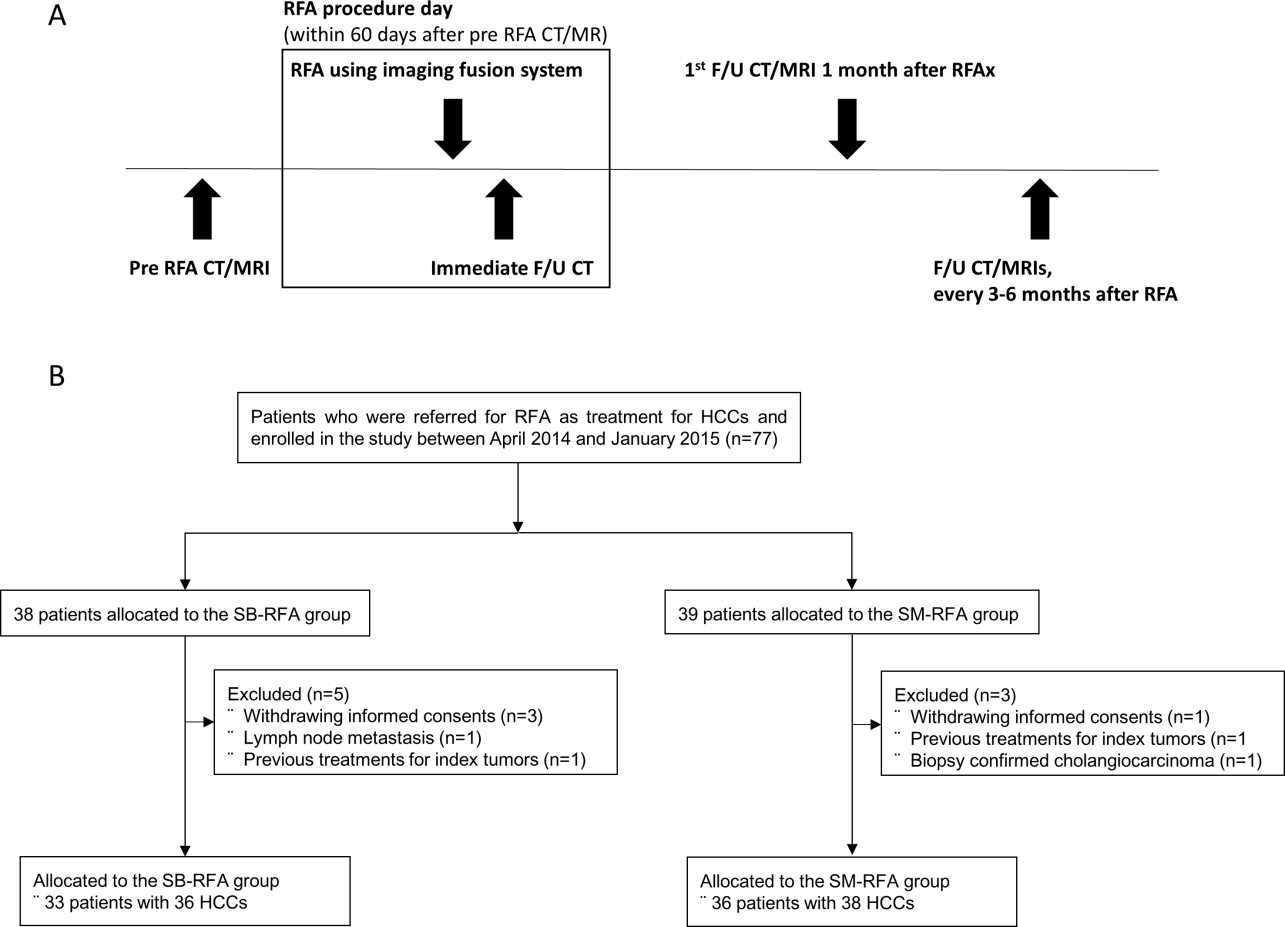
(A) Study protocol of the RFA procedure and intervals between the pre-RFA study, treatment, and follow-up. (B) Flowchart showing the consequences of the study flow. RFA, radiofrequency ablation; HCC, hepatocellular carcinoma; SB, switching bipolar; SM, switching monopolar, F/U, follow-up.

Among the 77 enrolled patients, 8 patients were excluded from the study for the following reasons: (a) patients withdrew their informed consent prior to RFA treatment (n = 4); (b) LN metastasis diagnosed on immediate post-RFA CT scan (n = 1); (c) previous treatment for the index tumor revealed after RFA treatment (n = 2); and (d) biopsy confirmed cholangiocarcinoma (n = 1). Finally, 69 patients with 74 HCCs comprised our study population. Thirty-three patients with 36 HCCs and 36 patients with 38 HCCs were included in the SB-RFA and SM-RFA groups, respectively (Fig 1). Additionally, tumors were subclassified according to tumor size into small (1~2.5 cm) and medium (≥2.5 cm) HCC groups for subgroup analyses. Baseline characteristics of all of the study patients are summarized in Table 1.

**Table 1:** Baseline characteristics of 69 patients with HCCs treated with radiofrequency ablation.

	Overall	SB-RFA	SM-RFA	p-value
M/F ratio	52/17	24/9	28/8	0.781
Age (mean, range, years)	61.4 (33~75)	60.3 (33~75)	62.4 (40~75)	0.36
Child-Pugh: A/B	68/1	33/0	35/1	1.000
Tumor size	2.00 ± 0. 69	1.99 ± 0.54	2.02 ± 0.42	0.894
Etiology of HCC(HBV/HCV/alcoholic/none)	52/9/4/4	27/3/1/2	25/6/3/2	0.655
Number of tumors(single/two)	64/5	30/3	34/2	0.275
Serum AFP(mean ± SD, ng/mL)	25.7 ± 67.1	38.4 ± 88.5	12.9 ± 31.1	0.125
Serum PIVKA(mean ± SD, ng/mL)	30.9 ± 32.6	33.0 ± 42.5	28.8 ± 19.3	0.632

Note.—SM = switching monopolar, SB = switching bipolar, RFA = radiofrequency ablation, HCC = hepatocellular carcinoma, HBV = hepatitis B virus, HCV = hepatitis C virus, AFP = alpha-fetoprotein, PIVKA = protein induced by vitamin K absence/antagonist-II

HCCs were diagnosed using one of the following criteria: (1) liver biopsies with pathologic confirmation (n = 8) or (2) typical imaging features as described by The American Association for the Study of Liver Diseases (AASLD) guidelines [2, 25] or Liver Imaging Reporting and Data System (LI-RADS)[26] (n = 66). HCCs were classified as perivascular tumors, if the index tumor had any contact with the first- or second degree branches of a portal or hepatic vein that was 3 mm or greater in axial diameter [27].

### RFA procedure

One experienced radiologist (J.M.L) with over 2000 cases of experience in image-guided RFA, performed all RFA procedures with one clinical fellow or senior radiology resident. Intravenous procedural sedation was induced for the ablation procedure using fentanyl citrate (Hana Pharm, Seoul, Korea), Midazolam (Hana Pharm, Seoul, Korea), and Ketamine (Huons, Hwaseong, Kyunggi, Korea) administered by a special nurse anesthetist with continuous monitoring of vital signs. After disinfection of the skin above the upper abdomen, local anesthesia of 2% lidocaine hydrochloride was applied to the region of electrode insertion. The electrodes were percutaneously inserted under ultrasound guidance with a real-time fusion of ultrasonography (US) and CT or MR images.

**Fig 2:**
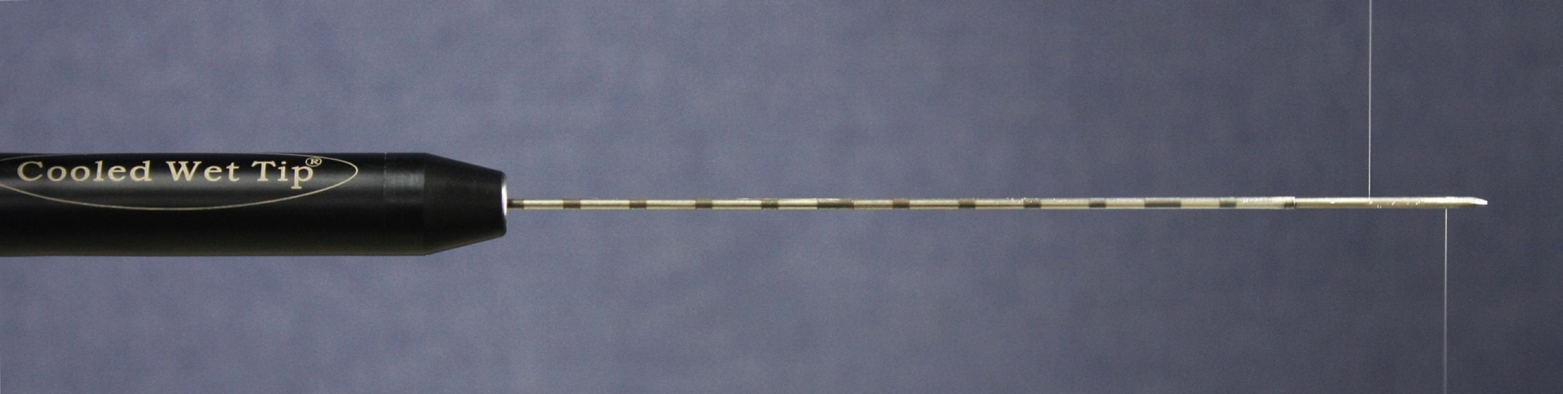
Photograph of an internally cooled wet electrode with two tiny (0.02 mm) side holes in the active tip.

In the SB-RFA group, three 17-gauge ICW electrodes and a multichannel RF generator (M-3004; RF Medical Co., Seoul, Republic of Korea) were used. The ICW electrode used in our prospective study contains two tiny side holes (0.02 mm in diameter) at the active tips in which less than 1% of the perfused normal saline remains within the electrode for electrode cooling which is then infused into tissue through these holes, at a rate of approximately 1 cc/min, (Fig 2). Sterilized, chilled, 0.9% isotonic saline was used for cooling and tissue perfusion.

In the SM-RFA group, a separable clustered electrode with three internally cooled electrodes (Octopus RF Electrode, STARmed, Goyang, Kyunggi, Korea), and another multichannel RF generator (Viva RF System, STARmed) were used. Each generator has a maximum power of 200W and performs automatic switching of RF energy among the three electrodes according to impedance changes in an electrode or a pair of electrodes. In both groups, the tip temperature of the electrodes was maintained at a range of 10–20°C by perfusing chilled 0.9% isotonic saline in the electrodes using peristaltic pumps (RFP-300; RF Medical Co., Seoul, Korea and VIVA Pump; STARmed, Goyang, Korea).

The prices of all RFA electrodes are set at the same price, according to the insurance policy of Korea, so the treatment cost of approximately 2000$ for each patient was same in both treatment groups.

**Fig 3:**
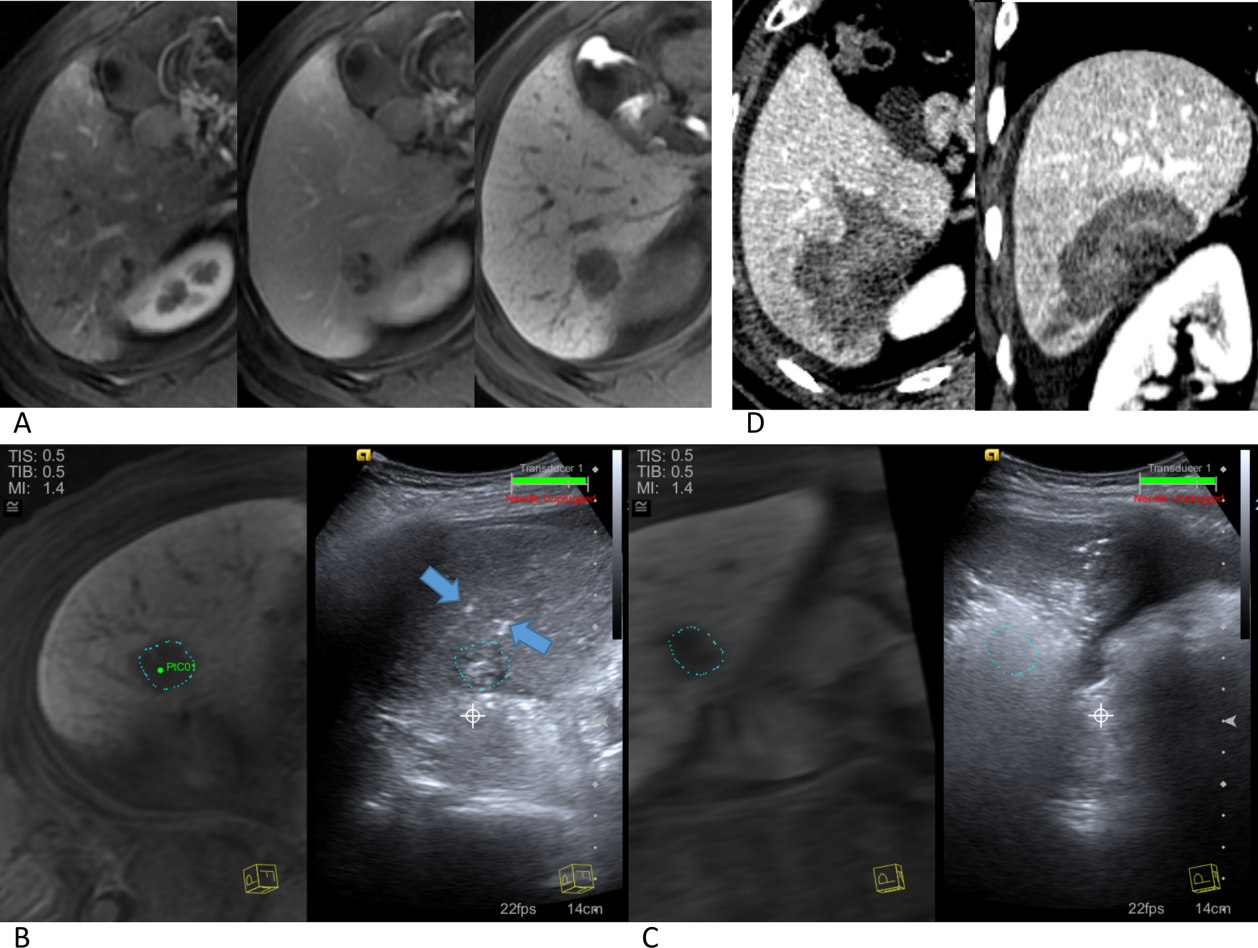
HCC in a 62-year-old man. (A) Axial MR images during arterial phase, portal phase and hepatobiliary phase after administration of gadoxetic acid show a 2.6 cm HCC with definitive arterial hypervascularization, venous washout, and hepatobiliary phase hypoenhancement. (B) Real-time US/MRI fusion image before ablation shows a slightly hyperechoic HCC on US image with virtual tumor margin and two electrodes (arrows) placed in the tumor and in the peritumoral area, respectively. (C) PostRFA US/MRI fusion image demonstrates that the virtual tumor margin suggesting the tumor location is covered by hyperechoic ablation zone with sufficient peritumoral margins. (D) Axial (left) and Coronal (right) immediate post-RFA CT images show complete ablation of the target tumor with sufficient peritumoral margins.

We chose the length of the active tip among 2.0 cm, 2.5cm, and 3.0cm according to the tumor size, shape, location and adjacent large vessels. If necessary, artificial ascites using a 5% dextrose solution was instilled to prevent adjacent organ injury to the subcapsular tumor or to improve the sonic window for the tumor not clearly visible on planning ultrasonography. For placement of the electrode in the target tumor, and for monitoring of the RFA procedures, real-time US-CT/MR fusion imaging systems (eSie Fusion: Acuson S3000, Siemens Healthcare, Erlangen, Germany; PercuNav: EPIQ 7, Philips, Best, Netherland) were used. In general, each of the three electrodes was placed in a triangle formation with an interelectrode distance of 1.5~2 cm. If a sufficient peritumoral margin was not created around the index tumor after one session of RF energy delivery for 8~12 minutes, additional ablations were performed after repositioning the electrodes. The RFA procedure was terminated when the hyperechoic ablation zone was considered to be sufficiently larger than the index tumor on US-CT/MR fusion imaging (Fig 3) [28]. Technical parameters such as the number of ablations, ablation time and total delivered energy were recorded for each target lesion. Additionally, a room occupying time, the time from patient in to patient out for the immediate post-RFA imaging study, was also recorded.

### Evaluation of treatment success, efficacy, and complications

#### Treatment success and efficacy

Immediately after RFA procedures, all patients underwent contrast-enhanced liver CT including arterial and portal venous phases in order to evaluate the technical success of the procedure and development of any possible complications. Technical success was defined as complete coverage of the index tumor by the ablation zone without any residual unablated tumors on immediate follow-up liver CT [10]. If technical success was not achieved, an additional RFA session was done immediately. Complete ablation with achievement of a sufficient peritumoral margin (>5 mm) was determined by the one of the authors with 6 years of experience in clinical imaging (W.C.) using a prototype temporal registration software (HepaCare, Siemens Healthcare) which performs non-rigid registration between the pre-RFA images (CT or MRI scans) and post–RFA CT images [9]. Technique efficacy was defined by the absence of nodular enhancement within or around the ablation zone on 1-month follow-up CT after the RFA procedure [10].

#### Measurements of ablation size and volume

Referencing previous studies [29, 30], the diameters and volume of the RFA-induced ablation zone were evaluated on immediate post-RFA contrast-enhanced, multiphasic liver CT scans which included unenhanced, arterial, portal venous, and 3-min delayed phase images. Maximum (Dmx), minimum (Dmi) and vertical (Dv) diameters were measured on the axial and coronal images of the portal phase which showed the largest ablation area showing no contrast enhancement. The volume of the non-enhancing ablation zone on CT was calculated with the assumption of the ablation zone as an ellipsoid using the following formula: ablation volume = π(Dmx x Dmi x Dv)/6 [10, 19, 31]

#### Complications

All procedure-related complications were recorded, and classified into major and minor complications according to the Society of Interventional Radiology Guidelines [10, 16]. In addition, any additional treatments given to the patients to manage the complications were also recorded.

### Evaluation of LTP, intrahepatic distant recurrence, and extrahepatic metastases

To evaluate the development of LTP or intrahepatic or extrahepatic metastases (EM), contrast-enhanced multiphase liver CT or MR was performed in all patients every 3~4 months following RFA treatment. Tumor recurrence was assessed in cases which achieved technical success and technique efficacy. The primary endpoint was LTP rate in both treatment groups. LTP was defined as the appearance of any new tumor foci showing arterial enhancement and washout on the portal or delayed scans at the site of the original tumor or adjacent to the ablation zone at follow-up scans [10, 16, 32]. Intrahepatic distant recurrence (IDR) was defined as the emergence of new HCCs in the liver which were not adjacent to the treated site [33, 34].

### Statistical analysis

All statistical analyses were performed using a statistics software IBM Statistical Package for Social Sciences 24.0 version (Chicago, IL, USA) and Excel 2016 version (Redmond, WA, USA). Continuous variables that could not pass the Shapiro-Wilk normality test were compared using the Z-test or Mann-Whitney test according to whether the number of each group was larger than 30 or not. Other continuous variables were compared using the t-test with unequal variances and Fisher’s exact test was used for the comparison of categorical variables. For the RFA parameters and ablation volumes, adjusted *P* values were calculated using Holm-Bonferroni Method to correct for multiple tests. The cumulative incidences of LTP, IDR and EM at 6, 12, and 24 months were evaluated using the Kaplan-Meier method with the log-rank test. A *P* value of less than .05 was considered to indicate a significant difference.

## Results

### Technical parameters and ablation volumes

The number of ablations was significantly lower (1.72 ± 0.70 vs 2.31 ± 1.37, p = 0.039) and ablation time was significantly shorter (10.9 ± 3.9 vs 14.3 ± 5.0 minutes, p = 0.004) in the SB-RFA group compared with the SM-RFA group. Delivered energy was also lower in the SB-RFA group (Table 2). Mean ablation volume was not significantly different between the two groups (Table 2). In addition, although sufficient peritumoral ablation margin greater than 5 mm was more often achieved in the SB-RFA group, no significant difference was observed (72.2% vs 55.3%, p = 0.153) (Table 2). Room occupying time in the SB-RFA group and the SM-RFA group were 59.7 ± 25.2 vs 64.7 ± 23.4 minutes, respectively (p = 0.393).

**Table 2:** Comparison of RFA variables and technique efficacy between SB-RFA and SM-RFA groups.

	Small HCC (1~2.5 cm)			Medium HCC (≥2.5 cm)			Overall		
	SB- RFA (n = 25)	SM-RFA (n = 26)	p-value	SB- RFA (n = 11)	SM-RFA (n = 12)	p-value	SB- RFA (n = 36)	SM-RFA (n = 38)	p-value
**Tumor size**	1.58 ± 0.46	1.66 ± 0.40	0.462	2.93 ± 0.40	2.78 ± 0.34	0.342	1.99 ± 0.54	2.02 ± 0.42	0.894
**No. of ablations**	1.64 ± 0.70	2.23 ± 1.39	0.432†	1.91 ± 1.22	2.75 ± 1.86	0.295†	1.72 ± 0.70	2.31 ± 1.37	0.039‡
**Ablation time (minutes)**	9.9 ± 4.0	13.0 ±5.0	0.058†	13.2 ± 5.1	17.3 ± 6.5	0.349†	10.9 ± 3.9	14.3 ± 5.0	0.004‡
**Energy (kcal)**	11.8 ± 6.4	22.2 ± 13.0	0.002†	16.3 ± 3.8	28.0 ± 11.9	0.016†	13.1 ± 6.3	23.4 ± 12.8	<0.001‡
**Ablation Volume (cm**^**3**^**)**	53.1 ± 19.5	50.6 ± 20.9	0.669	77.9 ± 25.6	64.6 ± 26.3	0.464	61.8 ± 24.3	54.9 ± 23.7	0.229
**Technical success**	100.0% (25/25)	96.2% (25/26)	1.000	100.0% (11/11)	100.0% (12/12)	1.000	100.0% (36/36)	97.4% (37/38)	1.000
**Technique efficacy**	96.0% (24/25)	96.0% (24/25)	1.000	100.0% (11/11)	100.0% (12/12)	1.000	97.2% (35/46)	97.3% (36/37)	1.000
**Sufficient peritumoral margin (≥5mm)**	80.0% (20/25)	57.7% (15/26)	0.132	54.5% (6/11)	50.0% (6/12)	1.000	72.2% (26/36)	55.3% (21/38)	0.153
**Major complication**	4.0% (1/25)	3.8% (1/26)	1.000	0.0% (0/11)	0.0% (0/12)	1.000	2.7% (1/36)	2.6% (1/38)	1.000
**Follow-up(mean ± SD, days)**	766.2 ± 211.5	739.0 ± 222.8	0.656	801.5 ± 98.7	751.6 ± 148.5	0.35	777.0 ± 210.4	764.3 ± 170.1	0.448

Note.—SM = switching monopolar, SB = switching bipolar, RFA = radiofrequency ablation, HCC = hepatocellular carcinoma

† Adjusted p-values of the Mann-Whitney test using Holm-Bonferroni Method

‡ Adjusted p-values of the Z-test using Holm-Bonferroni Method

### Technical success, technique efficacy, and complications

Among the 74 HCCs, 73 tumors were treated in a single session and one tumor in the SM-RFA group was treated in two sessions. Technical success was achieved in 100% (36/36) of cases in the SB-RFA group and 97.4% (37/38) of cases in the SM-RFA group. A residual tumor was noted on the immediate follow-up CT scan in the technical failure case of the SM-RFA group. The residual lesion was not visualized on planning ultrasonography confidently due to poor sonic windows, and was treated by transarterial chemoembolization (Table 2). Technique efficacy was achieved in 97.2% (35/36) and 97.3% (36/37) of cases in the SB-RFA and SM-RFA groups, respectively.

There were no RFA-related deaths observed in this study. Two major complications had occurred including one hepatic abscess requiring percutaneous drainage in the SB-RFA group (2.7%, 1/36) and one active bleeding from an intercostal artery requiring embolization in the SM-RFA group (2.6%, 1/38) (Table 2).

### Local tumor progression, intrahepatic recurrence, and metastases

#### LTP

**Fig 4:**
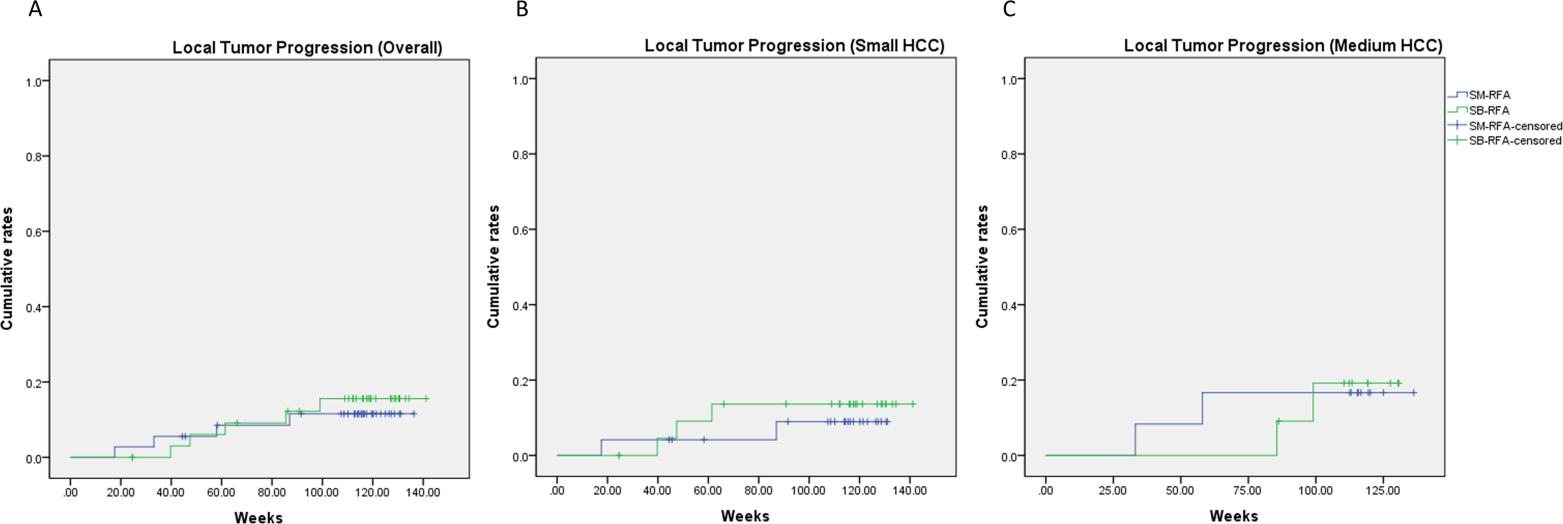
Cumulative local tumor progression rates after RFA of (A) overall, (B) small HCCs and (C) medium HCCs.

Mean follow-up periods were 777.0 ± 210.4 and 764.3 ± 170.1 days in the SB-RFA and SM-RFA groups, respectively. No deaths occurred during follow-up. The overall cumulative incidences of LTP at 6, 12 and 24 months were estimated as 0.0%, 6.1%, 15.7% in the SB-RFA group, and 2.8%, 5.6% and 11.6% in the SM-RFA group and were not significantly different between the two groups (p = 0.697) (Table 3, Fig 4). Subgroup analysis according to tumor size also did not reveal any significant differences. There were eight perivascular tumors (8/ 74, 10.8%): five in the SB-RFA group (5/36, 13.9%) and three in the SM-RFA group (3/38, 7.9%) (p = 0.4737). Among them, one case of LTP (1/8, 12.5%) developed in the SB-RFA group.

**Table 3:** Local tumor progression in 71 HCCs after successful RFA.

	Overall			Small HCC (1~2.5cm)			Medium HCC (≥2.5cm)		
Months	6	12	24	6	12	24	6	12	24
SB-RFA	0.0%	6.1%	15.7%	0.0%	9.1%	13.6%	0.0%	0.0%	19.2%
SM-RFA	2.8%	5.6%	11.6%	4.2%	4.2%	9.0%	0.0%	8.3%	16.7%
p-value		0.697			0.721			0.721	

Note.—SM = switching monopolar, SB = switching bipolar, RFA = radiofrequency ablation, HCC = hepatocellular carcinoma

#### IDR and EM

Among the 66 patients who achieved treatment success, IDR developed in 9 (9/32, 281%) and 17 patients (17/34, 50.0%) in the SB-RFA and SM-RFA groups, respectively.

**Fig 5:**
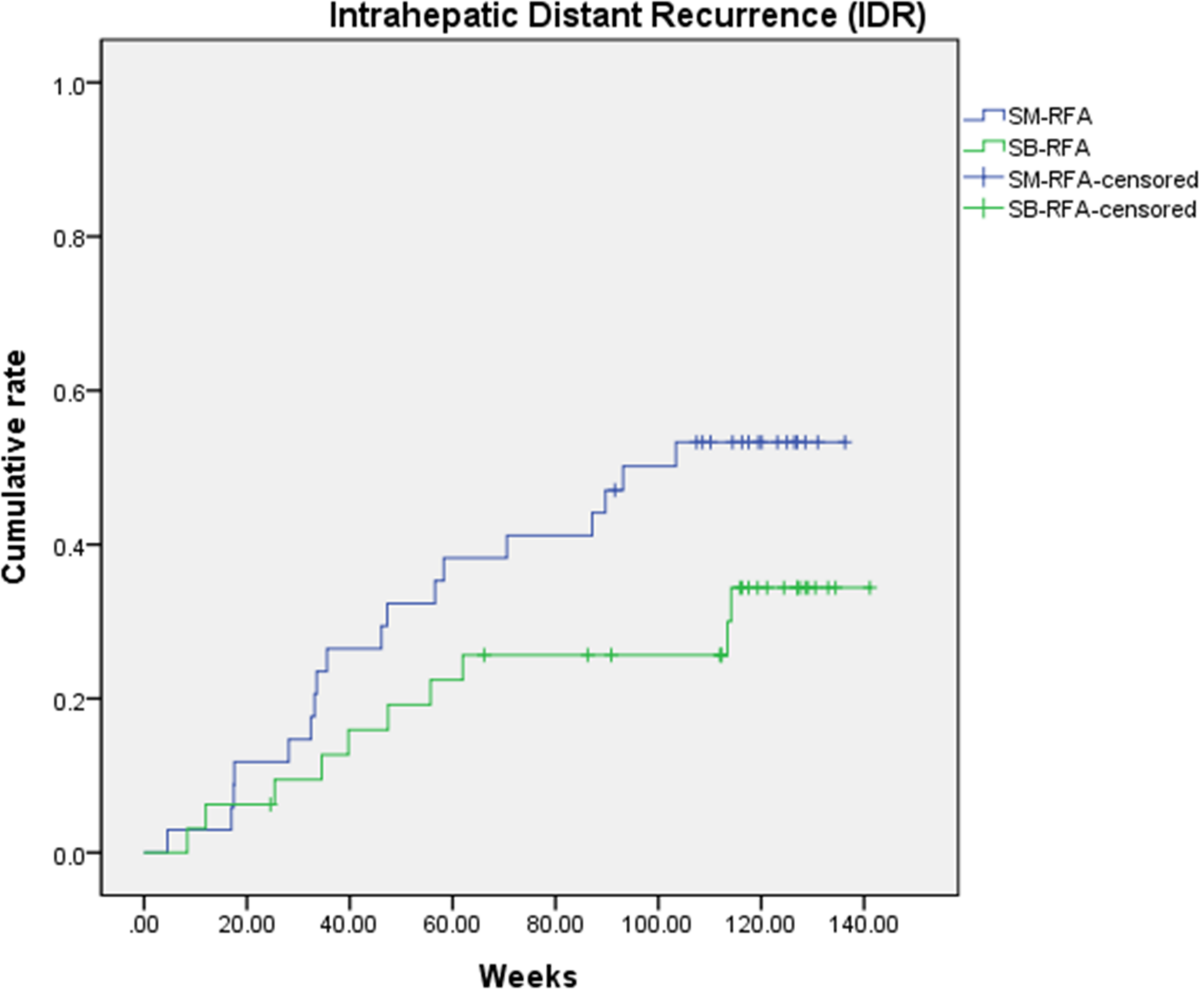
Cumulative intrahepatic distant metastasis after RFA.

The estimated cumulative incidences of IDR at 6, 12 and 24 months were estimated as 9.5%, 19.2%, 25.6% in the SB-RFA group, and 8.8%, 29.4% and 50.5% in the SM-RFA group (p = 0.114) (Fig 5). Extrahepatic metastasis occurred in only one patient of the SM-RFA group, 715 days after RFA treatment.

## Discussion

Our study demonstrated that both SB-RFA using ICW electrodes and SM-RFA using SC electrodes provided comparable LTP free survival rates although SB-RFA required fewer ablations and a shorter ablation time. Previous preclinical studies had demonstrated that SB-RFA could create a larger ablation volume at any given time compared to SM-RFA, and it was expected that SB-RFA would achieve an adequate peritumoral ablation margin and therefore would have a lower LTP compared with SM-RFA [17, 20]. However, according to our study results, the rates of achieved sufficient ablation margins around the target tumor were not significantly different between the two groups. The discrepancy between previous preclinical studies and our human study could be attributed to the use of a real-time US-CT/MR fusion imaging system for in situ monitoring of the ablation zone during RFA procedures in our study. With the use of a real-time US-CT/MR fusion imaging system, we could better monitor the three-dimensional relationship between the echogenic ablation zone and the index tumor during the RFA procedure, which would be difficult to achieve with B-mode US [35]. Thus, the operator was able to terminate the procedure when the echogenic ablation area covered the index tumor with greater confidence. As a consequence, even if SB-RFA could create a larger ablation zone per any given time compared to SM-RFA, the operator may terminate the procedure when the ablation zone was deemed sufficiently larger than the index tumor. Therefore, the ability to secure a sufficient peritumoral margin could be more dependent on the accuracy of the fusion imaging system rather than the difference of efficiency in creating larger ablation zones according to RF energy delivery mode, which probably would also lead to no significant difference in LTP. This is somewhat similar to previous meta-analysis results showing that both microwave ablation and RFA in the management of HCC had similar 1–5 year overall survival, disease-free survival, local recurrence rates and adverse events, although microwave ablation should have clear advantages over RF ablation, such as an improved convection profile, higher intratumoral temperatures, faster ablation times, larger ablation volumes, and less susceptibility to the heat-sink effect [36]. To the contrary, the shorter procedure time may indeed be beneficial for both patients and for the operator.

The technical success rates of our study were 100.0% and 97.4% in the SB-RFA and SM-RFA groups, respectively. All tumors were treated in a single session except for one tumor which had been treated in two sessions. The high technical success rates observed in both of our study groups could be attributed to the better efficiency of the multiple electrode approach, in either switching monopolar or bipolar modes, compared with conventional monopolar RFA using a single electrode in delivering RF energy to the target tissue, thereby allowing the creation of larger ablation zones [19, 29, 31, 37]. Another factor for the high technical success observed in our study could be attributed to the fact that compared with the multiple overlapping ablation approach using a single electrode which has great technical difficulty with US guidance, there was much less demand to relocate the electrode when multiple electrodes were used [38]. However, the cumulative 2 year LTP rates of SB-RFA and SM-RFA were still higher than 10%, which is much higher than the reported results of surgical resection [39]. This could be mainly attributed to the fact that a sufficient peritumoral ablation margin greater than 5 mm in three dimensions was achieved in 72.2% of tumors in the SB-RFA group, and 55.3% in the SM-RFA group. Indeed, the creation of a sufficient peritumoral margin in three-dimensions with RFA was quite challenging in several cases with advanced liver cirrhosis due to the poor sonic window, even under the guidance of a real-time US-CT/MRI fusion system [35]. Furthermore, registration accuracy of real-time US-CT/MRI fusion has been reported to be approximately 3 mm ~5 mm, and therefore, this misregistration could have resulted in the creation of an insufficient peritumoral safety margin [40]. Considering that the creation of an ablative margin of at least 5 mm is widely accepted to be one of the most important factors for reducing LTP in HCC after RFA [8, 39, 41], further improvement of the efficiency of multiple electrode RFA in creating large ablation zones and the development of an accurate in situ monitoring tool to better show the relationship between the index tumor and the ablation zone may further decrease the LTP after RFA.

Although bipolar RFA has better heat producing efficiencies, tissue dehydration and charring around the electrodes more commonly occurs in the bipolar mode than in the monopolar mode due to the higher current density around the electrodes, resulting in less efficiency in delivering RF energy to the target tumor [42]. In order to circumvent this problem, we used ICW electrodes for SB-RFA, which allow simultaneous internal cooling and interstitial perfusion of normal saline in our study [20]. Although internally cooled electrodes can preferentially decrease the heating of tissue nearest to the electrode, effectively preventing the charring of tissue in monopolar RFA [16], this technique does not sufficiently prevent overheating of the tissue around the electrode in the bipolar mode, which requires rapid switching of the active electrodes or elongation of the RF ablation time [23, 24]. In this regard, additional infusion of saline into the tissue can increase the electrical conductivity at the electrode-tissue interface and further decrease the heat generated by the electrical current flow through the tissue with high electrical impedance [43–46]. Furthermore, it can also increase thermal conductivity and the heat is more easily carried away from the electrode [17]. Our results of a lesser number of required ablations and shorter ablation times are largely owed to this ability of large and regular ablations using SB-RFA with ICW electrodes.

As for adverse events, both SB-RFA using ICW electrodes and SM-RFA using separable clustered electrodes showed low rates of complications (< 3%), similar to the results (4.1%) of a previous systematic review [47]. Theoretically, complication rates related to the RFA procedure can be increased in accordance with the number of electrode insertions, but in our study, safe and precise planning of the electrode insertion route, taking care to avoid bile ducts and major vessels, was possible under the guidance of fusion imaging, and the potential increase in complication rates related with the electrodes was able to be minimized. Furthermore, although there were no cases of skin burn in the SM-RFA group, SB-RFA has an advantage of not requiring grounding pads, and therefore, no risk of skin burns.

Recently, several studies [22, 48] showed the feasibility of RFA using the “no-touch” ablation technique with multiple electrodes in the multipolar mode, and also reported its promising outcomes including low LTP rates. The multiple electrode approach used in our study, especially in bipolar mode, can allow the “no-touch” ablation technique. In addition, considering that drainage vessels change in HCC from hepatic veins to peritumoral sinusoid or portal veins [49, 50], electrode insertion into the peritumoral area or peripheral portions of the target tumor can induce more extensive thrombosis in the draining peritumoral vessels which would be beneficial in decreasing the risk of intrahepatic metastases through the vessels. Indeed, in our study, the IDR rate was lower in the SB-RFA group, albeit without statistical significance. Experimental evidence that tumorogenic factors facilitating unwanted tumor recurrence may be produced after RFA treatment in the residual tumor and surrounding liver parenchyma have been raised up and these factors could differ in different modes of ablation[51–54]. And this might be another reason for lower IDR rate in the SB-RFA group. This phenomenon should be further evaluated in following studies.

There are several limitations to our study. First, this study compared the two groups using two different electrodes and two different RFA modes. Thus, compounding effects could not be completely avoided. Second, we did not perform the sample size calculation, and power analysis for this study and only a small number of patients were enrolled in our study for the explorative purpose. Therefore some results such as the differences in the LTP, rates of complete ablations, number of ablations in the subgroup analysis, and IDR were not confirmed in terms of statistical significance. Further study with a larger number of patients with longer follow-up periods is warranted.

In conclusion, both SB-RFA using ICW electrodes and SM-RFA using SC electrodes provided comparable LTP free survival rates although SB-RFA required less ablations and a shorter ablation time. In addition, as there were no clear differences between SB-RFA and SM-RFA, either technique can be favorably used for the percutaneous ablation of HCCs, depending on the operator’s familiarity and preference.

## Supporting information

S1 File CONSORT Checklist. (DOC)

S2 File Full study protocol in Korean. (DOC)

S3 File Summarized study protocol in English. (DOCX)

S4 File Anonymized raw data. (XLSX)
