# Supplementary material for: Comparison of switching bipolar ablation with multiple cooled wet electrodes and switching monopolar ablation with separable clustered electrode in treatment of small hepatocellular carcinoma: A randomized controlled trial
Source: PLoS One. 2018 Feb 8;13(2):e0192173. doi: 10.1371/journal.pone.0192173 (PMC5805261; doi:10.1371/journal.pone.0192173)
Supplement: S2 File — (DOC) [file pone.0192173.s002.doc]

**연구계획서**

**간세포암의 치료를 위한 냉각-식염수 주입 전극을 이용한 교대 이극성 고주파 열치료술: 예비 연구**

**Switching Bipolar Radiofrequency Ablation Using Cooled-Wet Electrode for Treatment of Hepatocellular carcinoma: a Preliminary Study**

**실시 기관명: 서울대학교 의과대학 영상의학교실**

**책임 연구자: 영상의학교실 교수 이 정민**

개요

| 연구제목 | **간세포암의 치료를 위한 냉각-식염수 주입 전극을 이용한 교대 이극성 고주파 열치료술: 예비 연구**  **Switching bipolar Radiofrequency Ablation Using Cooled-Wet Electrode for Treatment of Hepatocellular carcinoma: a Preliminary Study** |
| --- | --- |
| 연구목적 | 이 연구의 목적은 간세포암환자에서 고주파 열치료술 시행시 치료 효과를 극대화하기 위하여 내부냉각-식염수 주입전극(Cooled-wet electrode)를 이용하여 식염수 증강 교대 이극성 고주파 열치료술 (Switching bipolar RFA: 시험군)을 시행하고, 기존의 본원에서 이용하던 Octopus electrode를 이용한 교대 단극성 고주파 열치료술 (switching monopolar RFA: 대조군)과 비교하여 이 시술의 안전성, 괴사 부피, 종양주변에 5mm이상의 안전연의 확보를 위한 시술성공율 (technical success rate), 및 early safety data 를 시술 후 6 month follow-up에서 구하고, 임상적 치료 효과 (preliminary clinical outcome results: 12 개월 후 국소재발율)를 시술 후 12개월에 평가하고자 하는 본연구를 위한 피험자수를 결정하고자 함. |
| 연구기관 | 서울대학교 의과대학 영상의학교실 |
| 연구책임자 | 서울대학교 의과대학 영상의학교실 교수 이정민 |
| 연구대상 | CT나 MRI상 간세포암으로 새로이 진단받은 환자 중 미세침습적 치료법의 하나로 고주파 열 치료를 고려하는 환자로 선정기준과 제외기준을 모두 만족시키는 환자 |
| 연구대상수 | 평가 가능한 피험자 70명의 탈락율 10%를 반영한 77명  ( 시험군 :35명, 대조군 :35명) |
| 연구기간 | IRB 승인일 ~ 2015.02.28: 피험자 등록 및 고주파 열치료술 시술  IRB 승인일 ~ 2016.08.31: CT/MRI 추적검사 및 자료분석 |
| 연구개요 | 고주파 열치료술은 간세포암 및 전이성 간암의 효과적인 비수술적 국소치료 방법으로 각광을 받고 있다. 하지만, 기존의 고주파 열치료술의 중요한 한계점은 수술적 치료에 비하여 더 높은 국소 재발율을 보인다는 것이며, 최근 국소재발율을 10%이하로 줄이기 위해서는 10mm 두께의 안전연을 형성하는 것이 추천되고 있으나, 단일 전극을 사용시 단위 소작당 소작면적이 2-3.5cm정도로 제한적이어서 2cm이상의 종양을 치료 시 여러 번 전극의 위치를 변경시켜야 하나 소작 중 발생하는 미세기포로 인하여 정확한 위치의 변경이 매우 어려워 충분한 안전연을 확보하는 완전소작의 가능성이 줄기 때문이다. 따라서, 이러한 제한점을 극복하기 위해서 다전극침(clustered electrode, separable clustered electrode)등을 이용한 교대고주파 열치료술이나 다극성 또는 단극성 고주파 열치료술이 시도 되고 있으며, 본원에서는 최근까지 서로 분리가 가능한 세개의 전극침을 가진 다전극인 OCTOPUS 전극 및 switching RF generator system을 이용하여 고주파 열치료술을 시행하여 3년 국소 재발율이 11~15%정도임을 확인 하였다. 하지만 이러한 국소 재발 수치는 현재 까지도 수술의 국소 재발율 (~3%)에 비하여서는 국소 재발율이 높은 편이다. 이러한 결과는 RFA시술 중 전극 주변으로 overheating에 의한 charring이 발생하여 하나의 전극에 전달할 수 있는 최대 고주파 에너지가 200W이하이기 때문으로 생각된다. 최근 이를 극복하기 위하여 시술 중 전극의 내부냉각과 함께 전극의 active surface에 있는 작은 micropore를 통해 1cc이하의 소량의 생리식염수를 주입하면서 cooled wet electrode가 국내 기술로 개발되어 국내외 병원에서 쓰이고 있으며, multiple cooled wet electrode와 switching bipolar mode를 이용하면 세개의 전극 중 두개의 pair 전극에 전류를 교대로 전달하는 것이 가능하고, 단위 시간당 더 큰 부피의 괴사병변을 종양을 포함하여 만들 수 있으리라 예상된다. 이에 CT나 MRI상 간에 3개 이하의 간세포암이 있는 환자로 고주파 열 치료술를 고려하는 환자를 대상으로 cooled wet electrode과 switching bipolar RF generator를 이용하여 고주파 열치료술을 시행하고, 현재 본원에서 사용하고 있는 switching RF generator와 Octopus 전극을 이용한 교대 단극성 고주파 열치료술 방법( 대조군)과 비교하여 단위시간단 소작 병변의 부피 및 합병증 발생 빈도, technical success rate, 및 12개월 후 국소 재발율을 평가하여, 추후 두 치료법을 비교하고자 하는 본 연구를 위한 피험자 수를 결정하고자 하는 예비 연구이다. 시술은 기존의 3개의 전극을 가진 Separable cluster electrode를 이용하여 switching monopolar RFA 시술을 하던 방식(대조군: switching monopolar mode with separable clustered electrode)과 유사하게 3개의 전극(시험군: Swicthing bipolar mode with three cooled wet electrodes) 을 고주파 에너지 전달 전에 종양 내에 설치하고, 동시에 두개의 전극에 RF 에너지를 전달하고, 30초 간격으로 또 다른 전극에 고주파 에너지를 교대로 전달하는 방식으로 시행한다. 시술 후 종양의 반응 평가는 시술 후 1시간 이내에 CT를 촬영하여 technical success를 평가한다. 또한 시술과 관련한 합병증의 발생 빈도 및 평균 시술 시간을 평가할 예정이며, 이후 1개월 후, 그리고 이후에는 3개월 간격으로 12개월 후까지 CT영상을 얻어 국소 재발 유무를 평가할 예정이다. |
| 기대효과 및  예상결과 | Cooled-wet electrode 및 교대 이극성 고주파 발생기를 이용한 고주파 열치료술은 기존의 교대 단극성 고주파 열치료술에 비교하여 더 짧은 시간에 더 많은 고주파 에너지를 종양에 전달 하여 더 큰 응고괴사를 유도할 수 있어 치료 후 국소 재발율을 감소시킬 수 있을 것으로 예상된다 |

| **Sponsor** | **㈜ RF medical** |
| --- | --- |

**목 차**

1. 연구 제목 ------------------------------------------- 5

2. 실시기관명 및 주소 ------------------------------------ 5

3. 연구책임자 및 담당자 --------------------------------- 5

3.1 연구책임자

3.2 연구담당자

4. 연구 배경 및 목적 ------------------------------------- 5

5. 연구 대상자 선정기준 ---------------------------------- 10

6. 예상 연구기간 ---------------------------------------- 14

7. 연구방법 및 내용 계획 ------------------------------------ 14

8. 유효성 평가방법 -------------------------------15

9. 피험자 안전보호에 대한 대책 -------------------------- 18

10. 참고 문헌 ------------------------------------ 20

{별첨1} 설명문 및 동의서

연구비 내역서

**1. 연구 제목:**

**간세포암의 치료를 위한 냉각-식염수 주입 전극을 이용한 교대 이극성 고주파 열치료술: 예비 연구**

**Switching Bipolar Radiofrequency Ablation Using Cooled-Wet Electrode for Treatment of Hepatocellular carcinoma: a Preliminary Study**

# 2. 실시기관명 및 주소

기관 명칭: 서울대학교병원

주소: (110-744) 서울특별시 종로구 대학로 101번지

# 3. 책임연구자, 공동연구자, 담당자의 성명 및 직명

| **책임 연구자: 이정민** | | | | | |
| --- | --- | --- | --- | --- | --- |
| **역할** | **성명** | **소속기관 및 부서** | **직위** | **최종학위** | **전공** |
| **책임연구자** | **이정민** | **서울대학교 의과대학** | **교수** | **박사** | **영상의학** |
| 공동  연구자 및  담당자 | 윤 정환 | 서울대학교 의과대학 | 교수 | 박사 | 내과학 |
|  | 김 윤준 | 서울대학교 의과대학 | 부교수 | 박사 | 내과학 |
|  | 이 현희 | 서울대학교병원 영상의학과 | 연구간호사 | 학사 | 간호학 |
|  | 김 세형 | 서울대학교병원 | 부교수 | 박사 | 영상의학 |
|  | 이 재영 | 서울대학교병원 | 부교수 | 박사 | 영상의학 |
|  | 김 정훈 | 서울대학교 병원 | 부교수 | 박사 | 영상의학 |
|  | 이 동호 | 서울대학교 병원 | 전임의 | 석사 | 영상의학 |
|  | 서 경석 | 서울대학교 의과대학 | 교수 | 박사 | 외과학 |
|  | 이 정훈 | 서울대학교병원 | 조교수 | 박사 | 내과학 |
|  | 이 남준 | 서울대학교 의과대학 | 부교수 | 박사 | 외과학 |
|  | 윤 정희 | 서울대학교병원 | 조교수 | 석사 | 영상의학 |
|  | Nominzul Otgonjargal | 서울대학교병원 영상의학과 | 대학원생 | 학사 | 영상의학 |
|  | 장 원 | 서울대학교 병원 | 전임강사 | 석사 | 영상의학 |

**4 연구 배경 및 목적**

고주파 열치료술은 약 500 kHz내외의 교류성 전류를 종양에 가하여 조직 내 이온들의 떨림에 의한 마찰열을 발생시켜 조직에 응고성 괴사를 유발시키는 방법으로 간의 악성종양에 대한 치료법의 선택에 따른 예후는 논란이 많으나 최근 고주파 열치료술은 수술적 치료가 어려운 일차성 간암 및 이차성 간암의 치료를 위해 비침습적 국소 치료법의 하나로 널리 이용되고 있다(그림 1). 하지만, 기존의 고주파 열치료술의 중요한 한계점으로 거론되고 있는 것은 단위 소작당 소작면적이 2-3.5cm정도로 제한적 이다는 것이며, 이의 가장 큰 원인은 간 조직의 전기 전도도가 낮아서 전극 주변으로 고주파 에너지의 집중이 일어나고 이로 인하여 전극 주변 조직의 100oC이상으로 과열이 되고 최종적으로는 더 이상의 에너지의 전달이 불가능하게 된다. 따라서 현재의 고전적인 고주파 열 치료기기로는 종양과 종양 주변에 10mm 두께의 안전연을 형성하기 위해서는 2cm이상의 종양을 치료 시 여러 번 전극의 위치를 변경시켜야 하나 소작 중 발생하는 미세기포로 인하여 정확한 위치의 변경이 매우 어려워 충분한 안전연을 확보하는 완전소작의 가능성이 줄게 된다(그림 2).


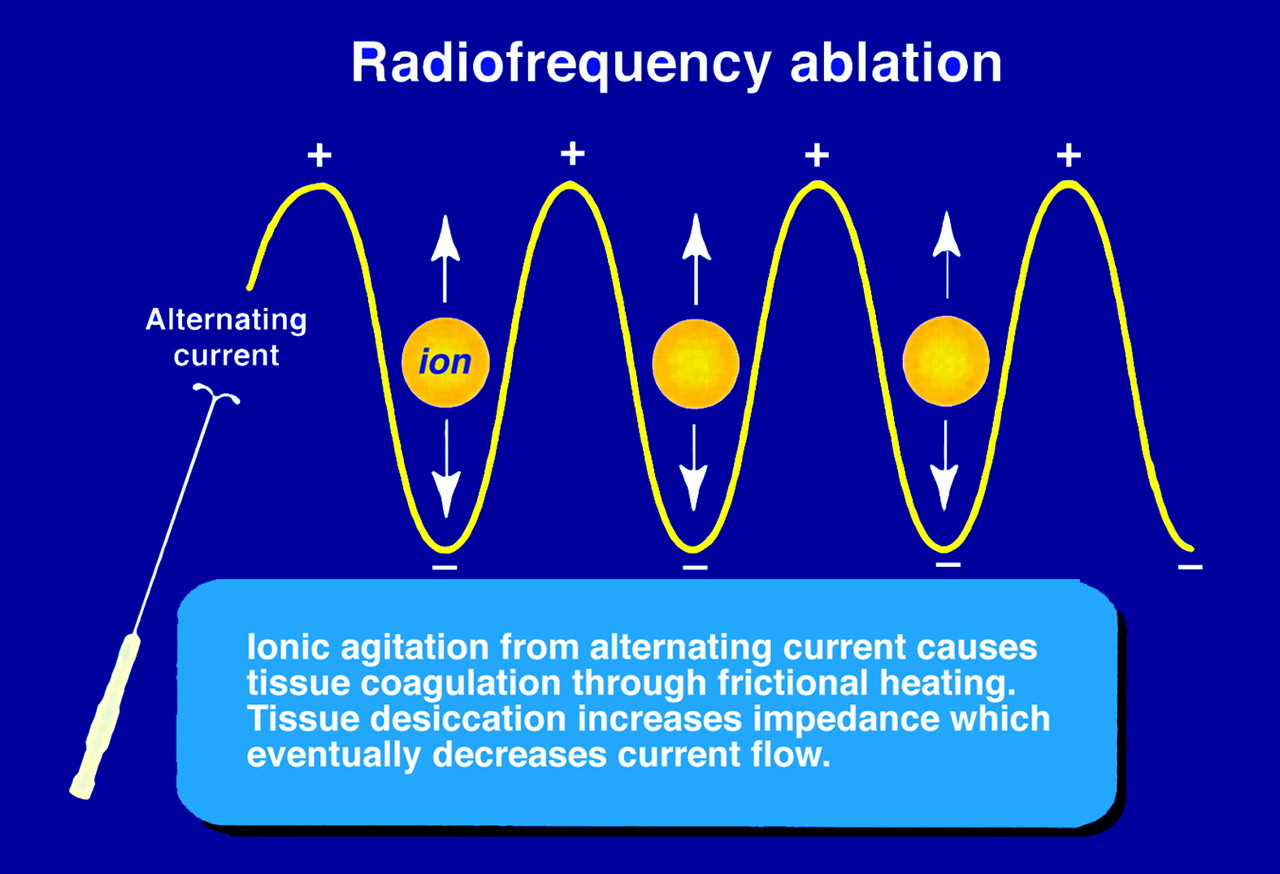

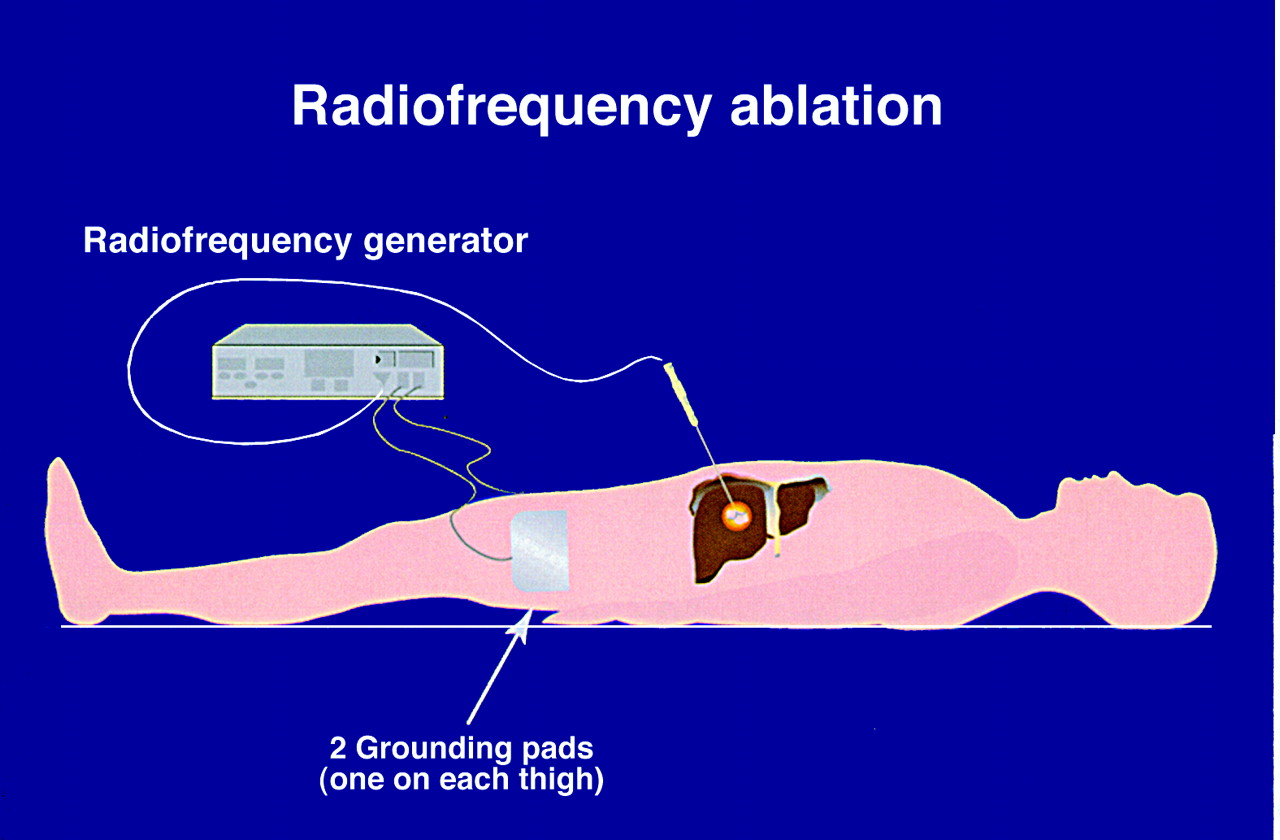


그림 1. 고주파 열치료술의 작동 기전 및 모식도


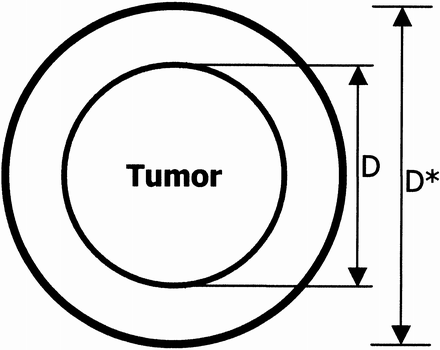


그림 2. 종양주변에 안전연 (safety margin)의 형성을 위한 overlapping ablation 의 필요성

현재 고주파 열 치료술에 가장 많이 사용되는 기기는 Monopolar RFA system으로 환자의 종양에 삽입한 고주파 전극과 generator 및 grounding pad가 하나의 회로를 이루는 system이며,  1회 치료 시 1개의 전극에만 고주파전류를 흘릴 수 있다 (그림 3).


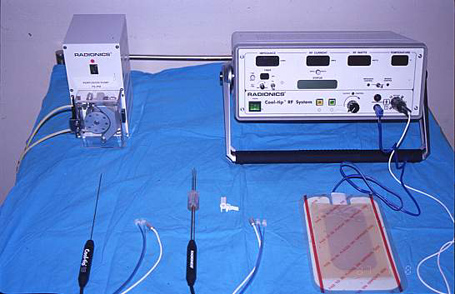


그림 3. Conventional RF generator 와 single, cluster electrode

따라서, 종양 주변으로 충분한 크기의 안전연 ( 5~10mm 두께)을 형성하기 위해서는 여러 번의 overlapping ablation이 필요하며, 이를 위해서는 치료 시간이 매우 증가하는 문제점이 있다. 이러한 치료 시간의 증가는 환자에게는 통증의 증가, 진통제의 과다사용 및 수술 중 고주파 열 치료를 시행하는 경우는 마취시간의 증가를 의미한다. 근래에 한번의 고주파 열 치료술로 좀 더 큰 응고괴사를 만들기 위하여 내부냉각 전극, 다침 전극, 및 우산 모양의 팽창형 전극 등의 새로운 전극의 개발, 및 보다 큰 RF power를 가진 고주파 발생기 등이 개발되었다.  각각의 방법은 기존의 고식적인 고주파 열 치료법에 비하여 단위 시간당 소작 범위를 증가시키는 효과가 있으나, 현재까지도 치료 대상이 되는 3-4cm크기의 간종양과 함께 종양주변의 정상조직을 10mm 정도 더 소작하기에는 소작범위가 작다. 이러한 새로운 전극의 개발에도 불구하고 현재까지의 monopolar RFA기법의 문제점으로는 고주파 열 치료 시 “종양을 태우기 위해 필요한 에너지의 양이 어떤 특정온도로 조직을 유지하는 데 필요한 에너지 보다 더 크다“는 점을 생각한다면 종양을 치료하기 위해 이용되는 고주파에너지의 대부분이 치료 초반에 이용되며 후반에는 고주파에너지가 비효율적으로 사용된다. 즉 Single channel RF generator를 이용한 고주파 치료방식은 active heating사이에 많은 dead time이 존재한다. 따라서 고주파발생기의 출력이 비효율적으로 사용되고 있다는 것이다.

2004년 Lim 등은 5cm 이하의 간세포암 환자 394명에 대해 고주파 열 치료를 시행한 결과 1, 3, 5년 생존율은 98%, 76%, 59%, 무병생존율은 70%, 29%, 20%를 나타냈다. 특히 Child-Pugh 분류 A, B군의 5년 생존율이 각각 67%와 34%로 Child-Pugh 분류가 생존율에 영향을 미치는 인자로 밝혀졌다. 2005년 Lencioni 등은 5cm 이하, 3개 이하의 간세포암 환자 187명에 대해 1, 3, 5년 생존율을 97%, 71%, 48%로 보고하였다. 2005년 Tateishi 등은 고주파 열 치료를 일차 치료로 시행한 319명 환자에서의 1, 3, 5년 생존율은 각각 95%, 78%, 54%로 보고하였다. 단일전극을 이용한 고주파 열 치료술의 제한점을 극복하기 위해 최근에는 본원에서는 switching RF generator와 분리형 클러스터 전극를 이용하여 고주파 열 치료술을 시행하고 있으며, 단위시간 당 고주파 에너지의 효율적인 전달 면에서 볼 때 2-3개의 고주파 전극을 교대로 전류를 흘려 주는 교대 고주파 열 치료방식은 단위시간당 고주파발생기의 효율적 사용을 의미한다. 본원에서는 현재까지 세개의 내부 냉각 전극 을 이용하거나 세개의 전극침을 가진 OCTOPUS 전극 과 Single switching RF generator system을 이용하여 고주파 열 치료술을 시행하여 3년 국소 재발율이 11~15%정도임을 확인 하였다. 하지만, 현재 까지도 수술의 국소 재발율 (~3%)에 비하여서는 국소 재발율이 높은 편이다. 이러한 결과는 단일 교대 고주파 열치료법이 시술 시간동안 고주파 에너지의 효율적인 전달이 가능 하기는 하나, 시술 도중 저항 수치의 증가함에 따라서 시간당 하나의 전극에 전달할 수 있는 최대 고주파 에너지가 150~200W이하로 감소하기 때문으로 생각된다. 이러한 현상의 발생 원인은 고주파술 도중 전극 주변의 고주파 에너지에 의한 overheating이 발생하여 조직이 charring이 발생하고 이로 인하여 저항 수치가 증가하여 높은 고주파 에너지를 줄수 없기 때문으로 추정된다.

최근 국내의 의료기기 제조사인 알에프 메디컬사는 내부냉각형 전극에 작은 micropore를 뚫어서 고주파 소작술 도중 치료하고자하는 조직에 1cc이하의 소량의 생리식염수를 전극 주변에 흘려줄 수 있는 내부냉각관류전극 (cooled wet electrode :제조 허가 08-891호) 를 개발하였다. 이러한 cooled wet electrode를 이용하면 고주파 치료 중 전극 주변의 charring을 막을 수 있어서 더큰 소작 병변을 만들 수 있는 것이 보고되었다 (Cha J et al. [Cardiovasc Intervent Radiol.](http://www.ncbi.nlm.nih.gov/pubmed/19488818) 2009 Nov;32(6):1235-40.). 또한 Internally cooled wet electrode를 이용한 임상연구에서 (Kim JH, et al, [J Vasc Interv Radiol.](http://www.ncbi.nlm.nih.gov/pubmed/23433411) 2013 Mar;24(3):351-6) 에서도 기존의 internally cooled electrode와 비교 시 (6-month and 1-, 2-, and 4-year local tumor progression rates: 11%, 24%, 32%, and 35%), 더 큰 소작 병변을 만들 수 있었으며, 낮은 local tumor progression rate (6-month and 1-, 2-, and 4-year local tumor progression rates: 5%, 13%, 15%, and 26%)를 보였다. 하지만, 이 역시 수술이나 switching monopolar RFA에 비해서는 유의하게 높은 국소 재발율이다. 따라서, 만약 세 개의 cooled wet electrode와 multichannel generator를 사용하여 saline-enhanced switching bipolar RFA를 시행한다면 단위 시간당 보다 효율적으로 더 큰 소작 병변을 만들 수 있으리라 기대된다. (그림 4). 따라서 기존의 single switching RF generator와 비교 시 단위시간당 두 개의 전극에 동시에 고주파 에너지를 전달 할 수 있으므로 단위시간당 고주파 에너지의 전달 효율이 증가하게 되며, 두 전극에 고주파 전류의 전달이 가능하게 되어 단위시간당 더 큰 소작 병변을 만들 수 있다 (그림 5). 이러한 cooled-wet electrode를 이용한 saline-enhanced switching bipolar RFA방식은 이전에 사용하던 single switching system에 비해서도 단위시간당 고주파 에너지의 열전달 효율이 향상되어 시술시간이 짧아지고 더 큰 종양 주변 안전연을 안정적으로 형성하여 국소 재발율을 줄일 수 있을 것으로 기대된다.


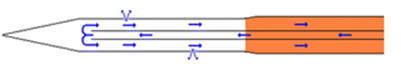

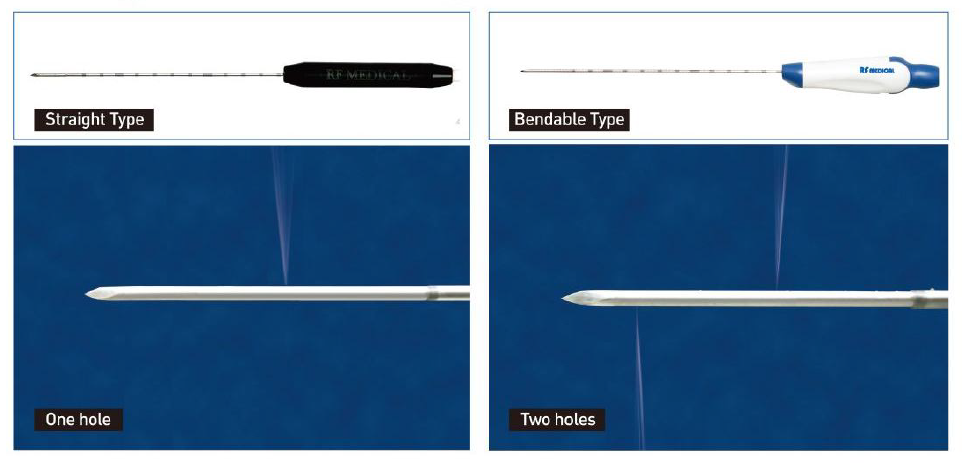
 
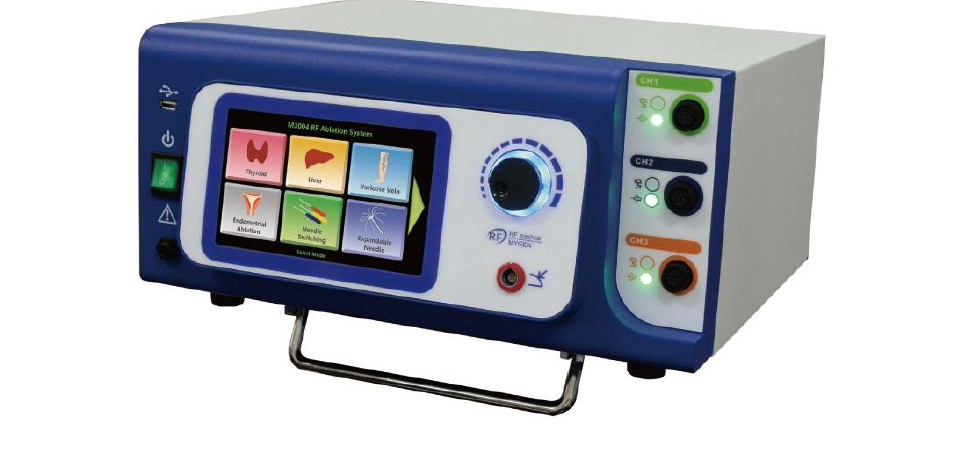


그림 4 Cooled wet tip 과 Multichannel generator (M-3004; 알에프 메디컬, 서울, 한국)


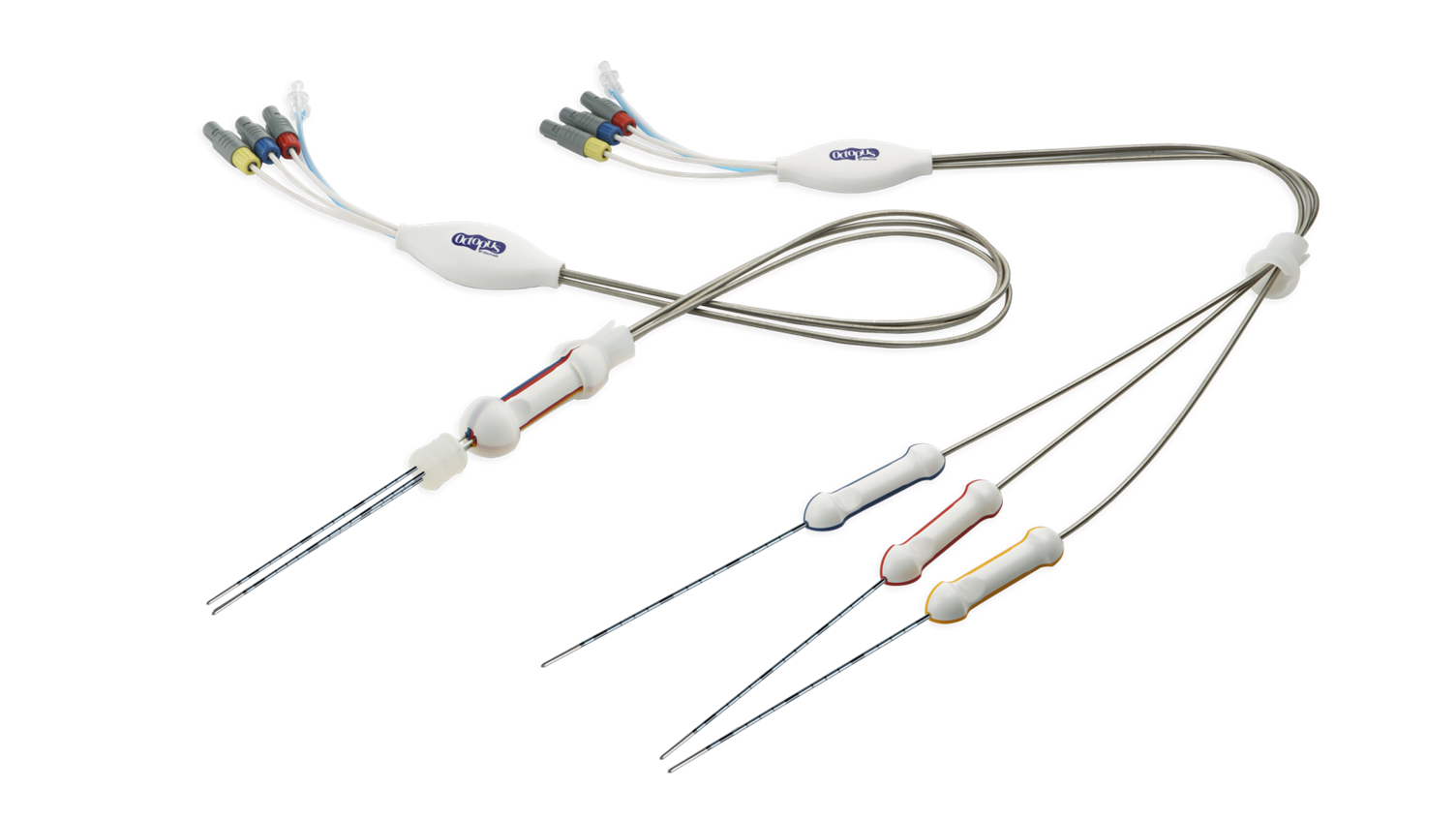
Octopus electrode ( 스타메드사)

(A)
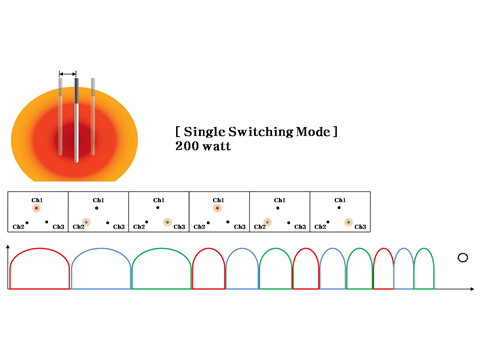


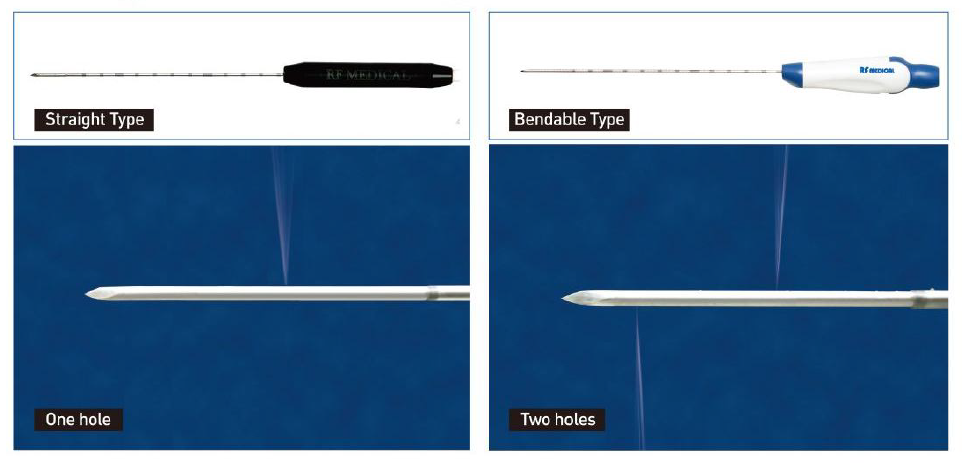
Cooled wet electrode (알에프 메디컬)


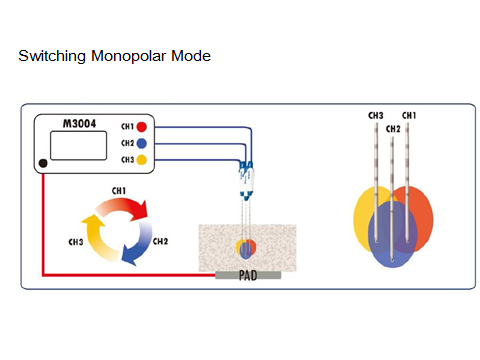


(b)

그림 5. (a) Single switching RF generator와 separable cluster 전극을 이용하여 3개의 전극 중 1~2개의 전극에 에 교대로 고주파 에너지를 전달할 수 있는 Switching monopolar RFA 중 얻은 RF energy instillation의 양상을 보여주는 그림 과 (b) switching RF generator와 cooled-wet 전극을 이용하여 3개의 전극 중 2개의 전극에 교대로 고주파 에너지를 전달할 수 있는 Switching bipolar RFA 중 얻은 RF energy instillation의 양상을 보여주는 그림

본 연구자들은 기존의 다 전극을 이용한 단일 교대 단극성 고주파 열 치료술의 국소 재발율이 11~15%으로 수술적 치료법에 비하여 높게 보고되고 있는 점을 고려할 때 cooled-wet 전극 및 교대 이극성 RF mode를 이용한 식염수 보강 고주파 열 치료술은 기존의 다 전극을 이용한 단일 교대 단극성 고주파 열 치료술에 비교 하여 치료성적의 향상을 가져올 수 있을 것으로 기대되며, 여러 연구에서 간세포암환자에서 경피적 고주파 열 치료술 후 완전괴사의 획득이 nonsurgical HCC를 가진 Child-Pugh Class A와 B 간경변증 환자의 생존율을 유의하게 증가시킨다는 것이 확인된 점을 고려할 때 궁극적으로 cooled-wet 전극 및 교대 이극성 RF mode를 이용한 고주파 열 치료술로 간암의 초기 완전괴사의 획득 성공율을 향상시킬 수 있다면 생존율의 향상을 기대할 수 있을 것으로 기대된다.

이에 상기 기술한 바대로 이전 다전극을 이용한 RFA 및 내부냉각 식염수 주입 전극(cooled-wet electrode)를 이용한 임상 연구의 자료 및 임상경험을 바탕으로 간암환자에서 이에 CT나 MRI상 간에 3개 이하의 간세포암이 있는 환자로 고주파 열 치료술을 고려하는 환자를 대상으로 cooled wet electrode과 multichannel RF generator(M 3004; RF medical Co.)를 이용하여 식염수 보강 교대 이극성 고주파 열 치료술 (Saline enhanced, switching bipolar RF ablation)을 시행하고, 현재 본원에서 사용하고 있는 switching RF generator (Starmed Ltd.)와 Octopus 전극을 이용한 교대 단극성 고주파 열 치료술 방법( Switching monopolar RFA, 대조군)과 비교하여 단위시간 당 소작 병변의 부피 및 합병증 발생 빈도, technical success rate, 및 12개월 후 국소 재발율을 평가하여, 추후 두 치료법을 비교하고자 하는 본 연구를 위한 피험자 수를 결정하고자 하는 예비 연구를 시행하고자 한다.

**5. 대상 질환 및 대상자**

**(1) 임상시험계획(study design)**

CT나 MRI상 간에 3개이하의 간세포암이 있는 환자들 중 고주파 열 치료술를 고려하는 환자를 대상으로 두 그룹으로 나누어 한군에서는 현재 본원에서 기본적으로 사용하고 있는 기존의 옥토퍼스 전극을 이용한 다채널 고주파 열치료술 (대조군: switching monopolar RFA군)을 시행하고, 다른 한군에서는 cooled wet 전극과 switching bipolar mode를 이용하여 고주파 열치료술을 시행한다 (시험군: Switching bipolar RFA 군). 이후 두 치료법에서 단위 세션당 ablation volume, technical success rate, short-term recurrence rate, average procedure time을 알아보고자 하는 Randomized 연구이다.

이 연구에는 서울대학교병원에 내원한 환자 중 내과 및 외과 전문의의 의학적 판단으로 영상의학과 에 고주파 열 치료술을 받기 위해 의뢰된 환자 중 연구의 목적을 이해하고, 연구에 참여하기로 결정한 총 70명의 환자가 포함될 예정이며, 이 들 환자를 대상으로 전향적으로 다채널 고주파 발생기와 옥토퍼스 전극를 이용한 고주파 열 치료술을 시행하여, 단위 세션당 소작부피, 평균 시술시간, 시술 후 조영증강 CT 상 조영증강이 되지 않는 부위가 종양 및 5mm의 안전연을 만들었는지에 관한 고주파 열치료술의 기술적 성공율, 합병증 및 12개월 후 국소 재발율 등을 평가하고자 한다. 이 들 변수 중 일차 유효성 변수는 12개월 후 DCE-CT 검사로 평가한 국소 재발율이다. 두 군에서 시험에서 얻은 평가 변수들을 비교 평가하여 본시험을 위한 피험자 수를 결정하고자 한다.

**(2) 대상 환자 군:** 연구 포함 조건을 만족하고, 연구에 필요한 동의서를 제출한, 평가 가능한 피험자70명이고, 이들을 대조군과 시험군에 각각 35 명씩 총 70명을 enroll하고자 한다. 기존의 보고에 따르면 고주파 열치료술의 치료 성적은 종양의 크기에 따라 성적이 다른 것으로 보고 되고 있어서 전체 70명의 환자를 종양의 크기(1~2.5cm 및 >2.5cm)를 고려하여 모집 하고자 합니다.

본 연구는 기존의 Octopus 전극을 이용한 교대 단극성 고주파 열치료술(대조군)과 내부냉각-식염수 주입 전극을 이용한 교대 이극성 고주파 열치료술(시험군)에 따른 12 개월 후 국소재발율의 차이를 탐색적으로 평가하는 연구이며 각 군에서 기대되는 국소재발율에 대한 근거가 부재하여 검정력 혹은 추정의 정밀성 확보를 위한 대상수 산출을 하지 않았다. 1년 동안 본원에서 CT나 MRI 상 간세포암으로 새로이 진단받은 환자 중 고주파 열 치료를 고려하는 환자는 약 150명 정도로 예상된다. 이를 근거로 목표 피험자 수는 연구에 동의하며 선정기준과 제외기준을 모두 만족하는 환자 70명(각 군 당 35명 씩)을 대상으로 하고자 한다.

총 모집 대상자수는 본 연구에 필요한 최소인원 70명의 탈락율 10%를 고려하여 총 77명으로 한다.

피험자는 시험군(Swicthing bipolar mode with three cooled wet electrodes)과 대조군(switching monopolar mode with separable clustered electrode)에 각각 무작위로 배정될 것이다. 연구대상자는 선정기준 및 제외기준에 따라 선별 후 동의서가 획득되면 피험자의 번호가 부여되고 이 번호에 따라 시험군과 대조군으로 1:1의 비율로 배정될 것이다. 무작위배정표는 연구와 무관한 제 3자에 의해, block size를 4로 하는 blocked randomization method에 의해 작성된다. 또한 무작위 배정 시에는 종양의 크기(1~2.5cm 및 >2.5cm)를 고려한다.

1) 1~ 2.5 cm HCC군 (n=50)

2) > 2.5cm HCC군 (n=20)

무작위 배정은 미리 작성된 무작위배정표를 이용하여 웹 기반으로 할당하며, 서울의대/서울대학교병원 의학연구협력센터 (Medical Research Collaborating Center)에서 관리 및 운용한다.

포함 조건 (inclusion criteria):

1. 프로토콜의 요구조건에 동의하고 동의서를 제출한다.
2. 20세 이상-75세 이하
3. 간경변증이 있는 환자 중 60일 이내에 시행한 MDCT나 MRI상 1cm-5cm 크기의 간세포암이 의심되어 고주파 열 치료를 고려하는 환자
4. 치료 예정 병변(index tumor)에 대해 이전에 다른 국소 치료를 하지 않았어야 한다.

** 간세포암의 진단 기준

- 1. 위험인자(HBV 양성, HCV 양성, 간경변증 등)가 있으면서
  2. 전산화단층촬영(MD CT), 역동적 조영증강 자기공명영상촬영(dynamic MRI), 중 한가지 이상에서 간세포암에 합당한 소견을 보일 때
  3. 조직검사상 간세포암이 진단된 경우
  4. Primovist enhanced MRI상 동맥기에 조영증강이 보이고 간세포 특이기에 조영결손으로 보이는 경우

*배제 조건 (exlusion criteria):*

1. 악성 간종양의 개수가 3개 이상인 경우
2. 종양의 최대 크기가 5cm 이상인 경우 (미만성의 암종)
3. 종양이 5mm 이상의 중심부 간 문맥이나 간정맥에 유착된 경우
4. 심한 간부전 (Child-Pugh grade III)
5. 악성 간 종양에 의한 간 혈관의 침습이 있는 경우
6. 심한 coagulopathy (혈소판 80,000/mm^3^ 이하인 경우, INR이 50%이상 prolongation)
7. 다발성 간외 전이가 있는 경우
8. 연구목적에 맞는 적절한 자료를 얻을 확률이 매우 낮은 상황

Child-Pugh 분류

|  | No. of Points* | | |
| --- | --- | --- | --- |
| Factor | 1 | 2 | 3 |
| Bilirubin (mg/dL) | <2 | 2 - 3 | >3 |
| Albumin (g/dL) | >3.5 | 2.8 – 3.5 | <2.8 |
| Prothrombin time (INR) | <1.8 | 1.8 – 2.3 | >2.3 |
| Ascites | None | Slight | Moderate |
| Encephalopathy | None | Minimal | Advanded |

*Grade A, 5-6points; Grade B, 7-9points; Grade C, 10-15points

**6. 예상 연구기간**

**1)** IRB허가일 ~ 2015.02.28일: 피험자 등록 및 고주파 열치료술 시술

**2)** IRB허가일 ~2018.02.28일 까지 마지막 피험자의 추적관찰 종료 및 자료분석

**7. 연구방법 및 내용**

### 1) 대상자 선정

연구 개시일 이후 내과, 외과에서 간세포암으로 진단받고 고주파 열치료술을 받기 위해 영상의학과에 의뢰된 환자 상기한 선정조건을 만족하는 환자

2) 피험자 동의 확보

본 연구담당자는 연구에 참여하는 피험자에게 사전에 연구의 목적, 방법, 예상되는 결과 등에 대해 쉽게 이해할 수 있도록 설명하며 동의서에는 연구담당자 및 피험자 또는 보호자가 서명한다.

3) 무작위 배정 계획

피험자는 시험군 (Swicthing bipolar mode with three cooled wet electrodes)과 대조군(switching monopolar mode with separable clustered electrode)에 각각 무작위로 배정될 것이다. 연구대상자는 선정기준 및 제외기준에 따라 선별 후 동의서가 획득되면 피험자의 번호가 부여되고 이 번호에 따라 시험군과 대조군으로 1:1의 비율로 배정될 것이다. 무작위 배정표는 연구와 무관한 제 3자에 의해, block size를 4로 하는 blocked randomization method에 의해 작성된다. 또한 무작위 배정 시에는 종양의 크기(1~2.5cm 및 >2.5cm)를 고려한다.

무작위 배정은 미리 작성된 무작위 배정표를 이용하여 웹 기반으로 할당하며, 서울의대/서울대학교병원 의학연구협력센터 (Medical Research Collaborating Center)에서 관리 및 운용한다.

4) 치료 전 시술 계획:

Multiphasic 또는 perfusion CT나 Primovist MRI에서 종양의 위치를 확인 하고, 필립스사의 Liver analysis program상에서 RFA planning program을 이용하여 overlapping횟수 및 access route등을 결정하고, RFA 시술 시 최근 많이 이용되는 US-CT-MR fusion tool을 이용하여 시술 전 영상과 초음파 영상을 융합하여 초음파 영상에서의 종양의 위치가 시술 전 영상에서 발견된 종양이 일치하는 지를 평가하고 또한 시술 중 발생하는 에코버블의 분포와 종양의 위치가 일치하는 지를 실시간으로 평가 할 예정이다. 이때 사용될 초음파 융합기기는 Esaote, Phillips, GE, 또는 Siemens사의 navigation system들이 이용될 예정이다. 이 fusion된 영상을 통해 종양에 설치할 전극의 위치 및 전극의 개수 그리고 안전한 접근 경로 등을 계획한다. 만약에 일반적으로 사용되는 CT나 MRI상 종양의 종영증강이 subtle하거나 종양의 extent가 잘 보이지 않거나 종양의 근처로 AP shunt와 같은 조영증강이 combine된 경우 Perfusion CT를 시행하여 종양의 extent를 좀더 정확히 평가할 예정이다.

5) 고주파 시술

대조군에서는 고주파 열치료의 장비로는 200W Switching RF generator ( Viva Multi generator: Star Med, 한국), Octopus electrode 15 gauge 또는 17 gauge (Star Med, 한국), 그리고 ground pad와 pump for internal cooling 등을 사용한다. 시험 군에서는 RF medical사의 RF ablation system M3004 기기와 Cooled-wet electrode 3개 ( Jet tip) 15 gauge 또는 17 gauge를 grounding pad 없이 internal cooling하면서 사용한다.

시술 방법은 두군 모두 동일하며, 초음파 또는 전산화 단층촬영 유도 하에 electrode 중 임상적 필요에 따라 2-3개의 고주파 전극침을 종양에 위치시킨 후 식염수로 전극을 냉각시키며 dual switching RF generator를 이용하여 전극에 고주파를 가하여, 종양의 크기에 따라 약 10-30여분간 섭씨 90-100도로 온도를 지속시킨다. 단 시험 군 에서는 electrode의 active surface에서 두 개의 hole을 통해 saline이 1cc/10min정도 주입될 예정이다.

RFA 시술 시 최근 많이 이용되는 US-CT-MR fusion tool (Easy Fusion-siemens & Esaote, PercuNav-Phillips)을 이용하여 시술 전 영상과 초음파 영상을 융합하여 초음파 영상에서의 종양의 위치가 시술 전 영상에서 발견된 종양이 일치하는 지를 평가하고 또한 시술 중 발생하는 에코버블의 분포와 종양의 위치가 일치하는 지를 실시간으로 평가할 예정이다. 이때 사용될 초음파 융합기기는 Esaote, Phillips, Siemens상의 navigation system들이 이용될 예정이다. Fusion US 영상에서 이미 계획된 전극의 접근 경로를 통해 전극을 종양 내에 안전하게 설치한다. 이후 고주파에너지를 교대 이극성 혹은 단극성 모드로 3개 중 1~2개의 전극침에 전달한다.

6) 추적 관찰

- 기존에 clinical routine으로 시행하던 대로 고주파 열 치료 시술 직후 CT를 시행할 예정이며 Tumor의 complete necrosis 평가를 위해 Siemens사의 hepacare program을 이용하여 시술 전 CT/MR영상과 시술 후 CT영상을 registration하여 Tumor의 Total necrosis 가 이루어졌는지를 평가할 예정이다. 잔여 종괴가 확인되거나 불충분한 안전연이 확보되지 않은 경우 추가로 시술을 시행하고 이에 대한 평가를 한다.
- 또는 RFA시술 후 1개월에 AFP 또는 CEA, CBC, LFT와 CT를 시행한다.
- Follow up 방법도 기존의 방법과 동일하며, 시술 1개월 이후 매 3개월마다 혈액검사와 CT를 12개월 동안 3~4회 시행한다. 이는 대부분의 국소 재발이 12개월이내에 발생하는 점에 기인하며, 이를 기준으로 국소 재발율을 평가할 예정임.
- 이 연구의 최종시점은 시술 후 12개월 에 얻은 추적 검사 소견에 준한다 (단기 재발율 평가).
- 하지만, 이후에도 환자는 기존의 고주파 열치료술을 받은 환자의 routine follow-up protocol에 따라 3년까지는 CT 추적검사를 3-6개월 간격으로 시행할 예정임.

8. 유효성 평가:

일차 평가 변수: 고주파 시행 후 첫 12개월 동안 종양의 국소 재발율

이차 평가변수;

- - 1. CT상 nonenhancing ablation area의 최대 직경 및 volume
    2. 시술 후 1개월 후 CT에서 완전괴사의 획득 (technical success)
    3. 평균 시술시간 및 합병증 발생율
    4. 시술 후 12, 24, 36 개월 동안 원격 재발율 및 전이발생율/
    5. 시술 후 24, 36개월 동안 국소 재발율
    6. 시술 전 CT/MR-시술 후 CT간 registration tool을 이용한 치료 효과 평가의 정확성
    7. US-CT-MR fusion 영상의 registration 의 정확성 평가
    8. 전극의 두께에 따른 RF ablation 시술의 time당 소작 부피의 비교 : Octopus 15 gauge electrode와 17 gauge electrode간의 비교

1) 효능평가

(1) Technical Success: 고주파 열치료 후 치료 효과 판정

가. 조영증강 CT (dual energy mode)로 아래의 criteria로 치료 효과를 판정

- 완전반응(complete response): 고주파 열치료 후 1개월 내에 병변부위에 조영증강이 없거나 염증반응에 의하여 주위에 얇은 테두리의 조영증강이 나타날 때
- 불완전반응(incomplete response): 고주파 열치료 후 1개월 내에 병변부위에 결절형의 조영증강이 지속될 때

시술 후 1개월째 CT 검사에서 완전반응일 경우에는 정해진 기준에 따라 추적 관찰을 한다. 그러나 불완전반응인 경우에는 의료진의 토의 하에 추가 치료법을 결정한다.

나. CT raw data를 이용하여 조영증강을 보이지 않는 구역의 부피를 3D reconstruction software를 이용하여 구한다.

다. Siemens사의 Hepacare프로그램으로 종양주변에 충분한 안전연이 생겼는지를 평가하고 이의 정확도를 추후 12개월 후 재발율과 비교한다.

(2) 평균 시술 시간

시술에 필요하였던 시간을 의미하며, 이는 초음파 유도 하에 전극을 종양 내에 삽입하기 위한 시간으로부터 모든 전극을 인체로부터 제거한 시간을 의미한다.

### (3) 간내 재발의 정의와 치료

- 국소 재발(local recurrence): 치료받은 초기 병변부위로부터 2cm이내에 새로 생긴 병변
- 원격 재발(distant recurrence): 치료받은 초기 병변부위로부터 2cm이상 떨어져서 새로 생긴 병변

추적 관찰 도중 재발이 발견된 경우를 말하며, 치료는 의료진의 토의 후 환자의 상태에 대한 최적의 치료법을 결정하게 된다.

### (4) 간외 재발의 정의와 치료

추적 관찰 도중 폐, 부신, 뼈, 림프절 등 간 이외의 부위에서 재발하는 경우를 말하며, 치료는 의료진의 토의 후 환자의 상태에 대한 최적의 치료법을 결정하게 된다.

(5) 현재 본원의 Cooled-tip RF system을 이용한 고주파 열치료술을 받은 환자의 historical data (retrospective cumulative data)와 비교. 이때 종양의 크기에 따라 세분화하여 15-30mm군, 31-40mm군, 41-50mm군으로 나누어 비교 평가할 예정임.

# 2) CT검사:

시술 당일 고주파 열치료술 직전에는 사용하는 fusion system (S3000-Easefusion 또는 Philips Epiq-PercuNav)에 따라 외부에 marker를 붙이고 perfusion CT를 간부위만 얻고, 고주파 열치료술 직 후에는 동맥기영상과 문맥기(dual energy)CT를 이용하여 고주파 열치료술에 의한 괴사범위를 측정할 예정이며, 추후 1개월, 그리고 3년 까지 매 3~6개월간격의 regular follow up CT(dual energy CT) 혹은 Liver MRI를 시행할 예정임.

| **Post RFA CT Protocol** | | |  | |
| --- | --- | --- | --- | --- |
|  | **Arterial Phase** | **Portal Liver CT** |  |  |
|  | Recon1 | Recon1 |  | |
| **KV** | 80 | 80 |  | |
| **mAs** | mAs 300 | reference mAs 300 |  | |
| **reference mAs / D-dom /Z-dom/Acs** | on | On |  | |
| **Rotation   time (sec)** | 0.5 | 0.5 |  | |
| **Slice Thickness (mm)** | 3 | 3 |  | |
| **Pitch** | 1.5 | 0.891 |  | |
| **Resolution/Kernel** | Standard | Standard |  | |
| **Filter** | A | A(Soft) |  | |
| **Matrix** | 512*512 | 512*512 |  | |
| **Scan 반복 횟수** | 1 | 1 |  | |
| **Scan Coverage** | 20cm | 20cm |  | |
| **IV Contrast protocol** | 2-6cc/sec  during 30sec | |  | |

9. 안전성 평가

시술 및 수술 후의 합병증 유무 및 정도에 대한 평가를 시행한다. 평가방법은 Clavien system을 사용할 계획이다. Clavien system은 다음과 같다.

**Grade I**: minor, non-life threatening complications, no residual disability

**Grade II**: potentially life-threatening, no residual disability

**Grade IIa**: invasive procedures (operation or radiologic intervention) not required to manage complications

**Grade IIb**: invasive procedures (operation or radiologic intervention) required to manage complications

**Grade III**: associated with residual disability

# Grade IV: death as a result of a complication

**10. 통계 분석 계획**

1) 일차변수인 유효성 분석 계획: 대조군과의 차이 추정

비교군(Matched control group) 과 시험대상군 에서 고주파 시행 후 첫 12개월 동안 종양의 cumulative local recurrence rate(국소 재발율)를 Kaplan Mayer 생존분석법을 이용하여 각군에서의 효과를 Mantel-Cox 검사나 Breslow 검사법을 이용하여 검증한다. 이후 두군간의 효과의 차이를 추정하기 위하여 MedCalc 통계프로그램 을 이용하여 본 study의 sample size의 계산할 예정입니다.

2) 이차 평가변수들의 분석 계획 ;

가) CT상 nonenhancing ablation area의 최대 직경 및 volume : 종속변수의 정규성 분포 가정 만족 여부에 따라 모수방법( independent T-test) 또는 비모수적 방법(Wilcoxon rank sum test)으로 비교 분석

나) 시술 후 1개월 후 CT에서 완전괴사의 획득 (technical success): chi-square or Fisher’s exact test.

다) 평균 시술시간: 종속변수의 정규성 분포 가정 만족 여부에 따라 모수방법( independent T-test) 또는 비모수적 방법(Wilcoxon rank sum test)으로 비교 분석

라) 합병증 발생율: chi-squire or Fisher’s exact test.

마) 시술 후 12개월 동안 원격 재발율 및 전이발생율: Single switching RFA와 Dual bipolar RFA시술군에서의 원격재발율 및 전이 발생율을 계산하고, 이를 chi-squire or Fisher’s exact test로 비교 분석함.

바) 시술 후 24, 36개월 동안 종양의 cumulative local recurrence rate(국소 재발율)와 cumulative distant recurrence rate(원격 재발율)를 Kaplan Mayer 생존분석법을 이용하여 각군에서의 효과를 Mantel-Cox 검사나 Breslow 검사법을 이용하여 검증한다.

사) 시술 전 CT/MR-시술 후 CT간 registration tool을 이용한 치료 효과 평가의 정확성: 백분율로 계산 가능

아) Single switching RFA와 Dual bipolar RFA시술군 간 시간당 소작 부위 생성의 효율성 비교: 시술 시간 (분)당 생성할 수 있는 소작 부피 및 최대 소작 부피를 Independent T-test 또는 Wilcoson Rank Sum Test로 비교

# 11. 피험자 안전보호에 대한 대책

본 연구는 간세포암 또는 직장-대장암으로부터의 전이성간암을 가진 환자를 대상으로 고주파 열치료술 시행 시 치료 효과를 극대화하기 위하여 내부냉각-식염수 주입전극(Cooled-wet electrode)를 이용하여 식염수 증강 교대 이극성 고주파 열치료술 (Switching bipolar RFA: 시험군)을 시행하고, 기존의 본원에서 이용하던 Octopus electrode를 이용한 교대 단극성 고주파 열 치료술 (switching monopolar RFA: 대조군)과 비교하여 이 시술의 안전성, 괴사 부피, 종양주변에 5mm이상의 안전연의 확보를 위한 시술성공율 (technical success rate), 및 조기 안전성자료를 시술 후 6개월 추적 검사에서 구하고자 하는 것입니다. 옥토퍼스 전극과 내부냉각-식염수 주입전극은 국내 식약청의 사용허가를 받은 기기이며, 옥토퍼스 전극은 기존의 수입전극인 클러스터전극이나 내부냉각전극과 전반적으로 비슷하나 세 개의 전극침을 분리할 수 있다는 차이가 있으며, 각각의 전극에 개별적으로 고주파 전류를 독립적으로 전달할 수 있다는 장점이 있고, 내부냉각-식염수 주입전극은 소량의 냉각식염수를 종양 내에 주입할 수 있는 장점이 있다. 이들 전극을 이용하여 시술할 경우 전극의 비용의 기존의 수입전극 1개와 동일하여 전극의 추가 사용에 관련한 치료 비용의 상승이 없다. 또한 기존의 전극과 제형이 동일하여 기존의 치료법에 비교하여 시술 시 추가적인 위험성을 내포하고 있지는 않으나 치료에 사용되는 모든 의료기기가 그러하듯이 시술 중 또는 시술 이후에 합병증이 발생할 수 있다. 고주파 열 치료술은 는 사실 기존의 고주파 열 치료 기기를 이용한 고주파 열치료술의 historical data는 Clavien system을 이용한 안전성 평가에서 Grade IIb 의 합병증이 생길 확률은 2% 이하며, 만약 발생시 대부분은 출혈이나 감염으로 인하며 Radiologic intervention으로 residual disability 없는 치유될 수 있는 경우가 대부분입니다. Grade III 또는 IV 의 발생빈도는 문헌에 의하면 0.3%이하 입니다. 합병증 발생에 관해서는 환자에게 설명하고 동의서를 받는 과정에서 주지될 것이다. 합병증은 치료 과정에서 발생할 수 있는 내용이므로 발생할 경우 의료진은 그 치료에 만전을 기할 것이며, 연구에 참여한 피험자에게 특별한 보상을 하지는 않는다.

본 연구에 참여하기로 동의할 경우 두 군의 하나에 무작위배정 되어 고주파 열 치료술을 받게 되며, 피험자는 시술 전에 치료 과정에 대해서 그리고 추적 관찰하는 동안 환자의 상태에 대해서 의료진으로부터 상세한 설명을 들을 것이며, 피험자가 원하는 때에는 언제든지 연구 담당자로부터 설명을 들을 수 있다. 또한 피험자는 연구기간 중 언제든지 연구 참여에 대한 동의를 철회할 수 있다. 및 연구 도중에 피험자의 연구참여가 피험자의 동의가 없이도 연구자에 의해 중지되는 경우 및 해당사유, 피험자의 지속적인 참여의지에 영향을 줄 수 있는 새로운 정보가 수집되면, 즉시 피험자 또는 대리인에게 알려질 것이라는 사실에 대해서도 추가 기술하여 주시기 바랍니다.

1. 임상시험 계획서의 승인 및 수정

임상시험의 승인을 얻거나 승인 받은 임상시험을 변경 실시하고자 하는 경우, 임상시험 단계별로 계획서 또는 변경 계획서에 대하여 임상시험심사위원회의 승인을 받는다. 승인 이전에 피험자를 임상시험에 참여시킬 수 없다.

2) 연구계획의 숙지

시험책임자 및 담당자들이 연구계획을 정확히 분석 및 숙지하고, 대상 피험자의 문제점을 적극적으로 대응한다.

3) 임상시험의 동의

임상연구에 들어가기 전에 피험자에게 시험내용 및 시험기기의 효과, 이상반응 및 안전성에 대한 모든 사항을 설명한 후 피험자 자신이 자발적으로 본 연구에 참여하겠다는 동의서를 받고 연구에 들어간다. 피험자 또는 법정대리인이 동의서 서식, 피험자 설명서 및 기타 문서화된 정보를 읽을 수 없는 경우에는 공정한 입회자가 동의를 얻는 전 과정에 참석한다. 이 경우, 동의서 서식, 피험자 설명서 및 기타 문서화된 정보를 피험자 또는 법정대리인에게 읽어주고 설명한 후, 피험자 또는 법정대리인이 피험자의 임상시험 참여를 구두로 동의하고 가능하다면 동의서 서식에 서명하고 자필로 해당 날짜를 기재하게 한 다음, 공정한 입회자도 동의서 서식에 서명하기 전에 동의서 서식과 피험자 설명서 및 기타 문서화된 정보가 정확하게 피험자나 대리인에게 설명되었고, 이들이 해당사실을 이해하였다는 것과 동의를 얻는 과정이 피험자나 법정대리인의 자유 의사에 따라 진행되었다는 것을 확인하여야 한다.

4) 연구자에 의한 연구 중단

연구 도중에 피험자의 연구참여가 피험자의 동의가 없이도 연구자에 의해 중지되는 경우 및 해당사유, 피험자의 지속적인 참여의지에 영향을 줄 수 있는 새로운 정보가 수집되면, 즉시 피험자 또는 대리인에게 알려질 것입니다.

5) 피험자의 비밀유지

피험자의 신원을 파악할 수 있는 기록은 비밀로 보장될 것이며, 임상시험의 결과가 출판될 경우에도 피험자의 신원을 비밀상태로 유지한다. 구체적인 내용은 다음과 같다. 증례기록서 등 임상시험에 관련된 모든 서류에는 환자 이름이 아닌 피험자 식별코드(일반적으로 환자의 이니셜, 스크리닝 번호)로 기록하고 구분한다.

## 6) 임상시험 실시기관의 모니터링

피험자의 권리와 복지 보호, 보고된 임상시험 관련 자료가 근거문서와 대조하여 정확하고, 완전하며, 검증이 가능한지 여부 확인, 임상시험이 승인된 계획서 및 GCP의 규정에 따라 수행되는지의 여부 확인을 위하여 모니터링을 실시한다. 임상시험에 대한 모니터링은 RF medical사 직원의 정기적인 시험자 방문과 전화를 통해서 이루어 질 것이다. 방문시 모니터는 기본적으로 환자기록 원본, 시험기기 관리 기록, 자료 보관(연구 파일)등을 확인한다. 또한, 모니터는 임상시험 진행과정을 잘 살피고, 문제가 있을 경우 시험자와 상의한다.

# 7) 임상시험의 순응도와 protocol 위반에 대한 처리

본 임상시험에서 시험기기의 시술은 임상시험책임자 또는 담당자의 책임 하에 충분한 교육을 받고 시술되도록 하여야 한다. 본 임상시험 연구자(책임자)와 모니터는 protocol 위반이 발생하지 않도록 protocol에 대해 충분히 숙지하고 철저히 이행하여야 한다.

**11. 참고문헌**

1. Lencioni R, Cioni D, Crocetti L, et al. Early stage hepatocellular carcinoma in patients with cirrhosis: long-term results of percutaneous image-guided radiofrequency ablation. *Radiology* 2005; 234: 961-967.
2. Tateishi R, Shiina S, Teratani T, et al. Percutaneous radiofrequency ablation for hepatocellular carcinoma: an analysis of 1000 cases. *Cancer* 2005; 103: 1201-1209.
3. Gazelle GS, Goldberg SN, Solbiati L, Livraghi T. State of the art: tumor ablation with radio-frequency energy. *Radiology* 2000; 217:633-646.
4. Garcea G, Lloyd TD, Aylott C, Maddern G, Berry DP. The emergent role of focal liver ablation techniques in the treatment of primary and secondary liver tumors. *Eur J Cancer* 2003; 39:2150-2164
5. Goldberg SN. Radiofrequency tumor ablation: principles and techniques. *Eur J Ultrasound* 2001; 13: 129-147.
6. Goldberg SN, Dupuy DE. Image guided radiofrequency tumor ablation: challenges and opportunities-part I. *J Vasc Interv Radiol* 2001; 12: 1021-1032
7. Dodd GD, Frank MS, Aribandi M, Chopra S. Chintapalli KN. Radiofrequency thermal ablation: computer analysis created by overlapping ablations. *AJR Am J Roentgenol 2002;* 177: 777-782
8. Choi D, Lim HK, Kim MJ, et al. Overlapping ablation using a coaxial radiofrequency electrode and multiple cannulae system: experimental study in ex-vivo bovine liver. *Korean J Radiol* 2003; 4: 117-123
9. Livraghi T, Goldberg SN, Lazzaroni S, et al. Hepatocellular carcinoma: radiofrequency ablation of medium and large lesions. *Radiology2000;* 214: 761-768
10. Haemmerich D, Lee FT Jr, Schutt DJ, Sampson LA, Webster JG, Fine JP, et al. Large volume Radiofrequency ablation of ex vivo bovine liver with multiple cooled cluster electrode. *Radiology* 2005; 234: 563-568
11. Lee FT Jr, Haemmerich D, Wright AS et al. Multiple probe radiofrequency ablation: pilot study in an animal model. *J Vasc Interv Radiol* 2003; 14: 1437-1442.
12. Laeseke PF, Sampson LA, Haemmerich D et al. Multiple-electrode radiofrequency ablation creates confluent areas of necrosis: in vivo porcine liver results. *Radiology* 2006; 241: 116-124.
13. Lee JM, Han JK, Kim HC, et al. Switching monopolar radiofrequency ablation technique using multiple, internally cooled electrodes and a multichannel generator: ex vivo and in vivo pilot study. *Invest Radiol* 2007; 42:163-171.
14. Clasen S, Schmidt D, Dietz K, et al. Bipolar radiofrequency ablation using internally cooled electrodes in ex vivo bovine liver: prediction of coagulation volume from applied energy. *Invest Radiol* 2007;42:29-36.
15. Frericks BB, Ritz JP, Roggan A, Wolf KJ, Albrecht T. Multipolar radiofrequency ablation of hepatic tumors: initial experience. *Radiology* 2005; 237: 1056-1062.
16. Clasen S, Schmidt D, Boss A, et al. Multipolar radiofrequency ablation with internally cooled electrodes: experimental study in ex vivo bovine liver with mathematic modeling. *Radiology* 2006;238:881-890.
17. Lee JM, Han JK, Kim SH et al. A comparative experimental study of the *In-Vitro* efficiency of hypertonic saline-enhanced hepatic bipolar and monopolar radiofrequency ablation. *Korean J Radiol*  2003; 4: 163-169
18. Goldberg SN, Stein M, Gazelle GS, Sheiman RG, Kruskal JB, Clouse ME. Percutaneous radiofrequency tissue ablation: optimization of pulsed-RF technique to increase coagulation necrosis. *J Vasc Iinterv Radiol* 1999; 10: 901-916.
19. Liszezak TM, Hedley-Whyte ET, Adams JF, et al. Limitations of tetrazolium salts in delineating infracted brain. *Acta Neuropathol (Berl)* 1984; 65: 150-157.
20. Goldlust EJ, Placzynski RP, he YY, Hsu CY, Coldberg MP. Automated measurement of infarct size with scanned images of triphenyltetrazolium chloride-stained rat brains. *Stroke* 1996; 27: 1657-1662
21. Goldberg SN, Charboneau JW, Dodd GD 3^rd^, et al. Image-guided tumor ablation: proposal for standardization of terms and reporting criteria. *Radiology* 2003; 228: 335-345.
22. Chinn SB, Lee FT Jr, Kennedy GD, et al. Effect of vascular occlusion on radiofrequency ablation of the liver: results in a porcine model. *AJR Am J Roentgenol* 2001;176: 789-795.
23. Do Carmo MP. Differential geometry of curves and surfaces. Englewood, NJ: Prentice-Hall, 1976; 31-35Lau WY, Leung TWT, Yu SC, Ho SKW. Percutaneous local ablative therapy for hepatocellular carcinoma: a review and look into the future. *Ann Surg* 2003: 237; 171-179.
24. http://rsb.info.nih.gov/ij/, accessed November 20^th^, 2007-3-8
25. Haemmerich D, Tungjitkusolmun S, Staelin ST, Lee FT, Mahvi DM, Webster JG. Finite-element analysis of hepatic multiple probe radio-frequency ablation. *LEEE Trans Biomed Eng* 2002; 49: 836-842
26. Clasen S, Schmidt D, Boss A, et al. Multipolar radiofrequency ablation with internally cooled electrodes: experimental study in ex vivo bovine liver with mathematic modeling. *Radiology* 2006; 238: 881-890.
27. Bitsch RG, Dux M, Helmberger T, Lubienski A. Effect of vascular perfusion on coagulation size in radiofrequency ablation of ex vivo perfused bovine livers. *Invest Radiol* 2006; 41: 422-427.
28. Pereira PL, Trubenbach J, Schenck M, et al. Radiofrequency ablation: in vivo comparison of four commercially available devices in pig livers. *Radiology* 2004; 232: 482-490.
29. Komorizono Y, Oketani M, Sako K, et al. Risk factors for local recurrence of small hepatocellular carcinoma tumors after a single session, single application of percutaneous radiofrequency ablation. *Cancer* 2003; 97:1253-1262.
30. Hori T, Nagata K, Hasuike S, et al. Risk factors for the local recurrence of hepatocellular carcinoma after a single session of percutaneous radiofrequency ablation. *J Gastroenterol*. 2003;38:977-981.
31. Hines-Peralta AU, Pirani N, Clegg P et al. Microwave ablation: results with a 2.45-GHz applicator in ex vivo bovine and in vivo porcine liver. *Radiology* 2006; 239:94-102.
32. Haemmerich D, Laeseke PF. Thermal tumour ablation: devices, clinical applications and future directions. *Int J Hyperthermia* 2005;21:755-760.
33. Lee JM, Han JK, Kim SH, et al. Saline-enhanced hepatic radiofrequency ablation using a perfused-cooled electrode: comparison of dual probe bipolar mode with monopolar and single probe bipolar modes. *Korean J Radiol* 2004;5:121-127.
34. Solazzo SA, Ahmed M, Liu Z, Hines-Peralta AU, Goldberg SN. High-power generator for radiofrequency ablation: larger electrodes and pulsing algorithms in bovine ex vivo and porcine in vivo settings. *Radiology* 2007;242:743-750.
35. Pompili M, Mirante VG, Rondinara G, et al. Percutaneous ablation procedures in cirrhotic patients with hepatocellular carcinoma submitted to liver transplantation: Assessment of efficacy at explant analysis and of safety for tumor recurrence. *Liver Transpl*. 2005;11:1117–1126.
36. Lu DS, Yu NC, Raman SS, et al. Percutaneous radiofrequency ablation of hepatocellular carcinoma as a bridge to liver transplantation. *Hepatology*. 2005;41:1130 –1137.
37. Brillet PY, Paradis V, Brancatelli G, et al. Percutaneous radiofrequency ablation for hepatocellular carcinoma before liver transplantation: a prospective study with histopathologic comparison. *Am J Roentgenol*. 2006;186: S296–S305.
38. Llovet JM, Vilana R, Bru´ C, et al.; Barcelona Clínic Liver Cancer Group. Increased risk of tumor seeding after percutaneous radiofrequency ablation for single hepatocellular carcinoma. *Hepatology*. 2001;33:1124 –1129.
39. Livraghi T, Lazzaroni S, Meloni F, et al. Risk of tumor seeding after percutaneous radiofrequency ablation for hepatocellular carcinoma. *Br J Surg*. 2005;92:856–858.
40. Stigliano R, Marelli L, Yu D, et al. Seeding following percutaneous diagnostic and therapeutic approaches for hepatocellular carcinoma. What is the risk and the outcome? Seeding risk for percutaneous approach of HCC. *Cancer Treat Rev*. 2007;33:437– 447.
41. Poon RT, Ng KK, Lam CM, et al. Radiofrequency ablation for subcapsular hepatocellular carcinoma. *Ann Surg Oncol*. 2004;11:281–289.
42. Latteri F, Sandonato L, Di Marco V, et al. Seeding after radiofrequency ablation of hepatocellular carcinoma in patients with cirrhosis: a prospective study. *Dig Liver Dis*. 2008;40:684–689.
43. Huang GT, Lee PH, Tsang YM, et al. Percutaneous ethanol injection versus surgical resection for the treatment of small hepatocellular carcinoma: a prospective study. *Ann Surg*. 2005;242:36–42.
44. Livraghi T, Meloni F, Di Stasi M, et al. Sustained complete response and complications rates after radiofrequency ablation of very early hepatocellular carcinoma in cirrhosis: Is resection still the treatment of choice? *Hepatology*. 2008;47:82– 89.
45. Chen MS, Li JQ, Zheng Y, et al. A prospective randomized trial comparing percutaneous local ablative therapy and partial hepatectomy for small hepatocellular carcinoma. *Ann Surg*. 2006;243:321–328.
46. Lu MD, Kuang M, Liang LJ, et al. Surgical resection versus percutaneous thermal ablation for early-stage hepatocellular carcinoma: a randomized clinical trial. *Zhonghua Yi Xue Za Zhi*. 2006;86:801– 805.
47. Montorsi M, Santambrogio R, Bianchi P, et al. Survival and recurrences after hepatic resection or radiofrequency for hepatocellular carcinoma in cirrhotic patients: a multivariate analysis. *J Gastrointest Surg*. 2005;9:62– 67.
48. Hong SN, Lee SY, Choi MS, et al. Comparing the outcomes of radiofrequency ablation and surgery in patients with a single small hepatocellular carcinoma and well-preserved hepatic function. *J Clin Gastroenterol*. 2005; 39:247–252.
49. Lupo L, Panzera P, Giannelli G, et al. Single hepatocellular carcinoma ranging from 3 to 5 cm: radiofrequency ablation or resection? *HPB (Oxford)*. 2007;9:429–434.
50. Guglielmi A, Ruzzenente A, Valdegamberi A, et al. Radiofrequency ablation versus surgical resection for the treatment of hepatocellular carcinoma in cirrhosis. *J Gastrointest Surg*. 2008;12:192–198.
51. Taura K, Ikai I, Hatano E, et al. Implication of frequent local ablation therapy for intrahepatic recurrence in prolonged survival of patients with hepatocellular carcinoma undergoing hepatic resection: an analysis of 610 patients over 16 years old. *Ann Surg*. 2006;244:265–273.
52. Choi D, Lim HK, Rhim H, et al. Percutaneous radiofrequency ablation for recurrent hepatocellular carcinoma after hepatectomy: long-term results and prognostic factors. *Ann Surg Oncol*. 2007;14:2319 –2329.
53. Lu MD, Yin XY, Xie XY, et al. Percutaneous thermal ablation for recurrent hepatocellular carcinoma after hepatectomy. *Br J Surg*. 2005;92:1393–1398.
54. Yang W, Chen MH, Yin SS, et al. Radiofrequency ablation of recurrent hepatocellular carcinoma after hepatectomy: therapeutic efficacy on early and late-phase recurrence. *Am J Roentgenol*. 2006;186:S275–S283.
55. Cha J et al. Radiofrequency ablation zones in ex vivo bovine and in vivo porcine livers: comparison of the use of internally cooled electrodes and internally cooled wet electrodes. Cardiovasc Intervent Radiol. 2009 Nov;32(6):1235-40.
56. Kim JH, Kim PN, [Won HJ](http://www.ncbi.nlm.nih.gov/pubmed?term=Won HJ%5BAuthor%5D&cauthor=true&cauthor_uid=23433411), Shin YM. Percutaneous radiofrequency ablation with internally cooled versus internally cooled wet electrodes for small subphrenic hepatocellular carcinomas. J Vasc Interv Radiol. 2013 Mar;24(3):351-6

# [별첨 1] 연구수행요약표

|  | ScreeningVisit^1)^ | 시술 | 추적관찰기간^2)^ | | |
| --- | --- | --- | --- | --- | --- |
|  |  |  | 1개월 후 | 12개월 동안(3개월간격) | 12개월-3년 동안*  (3-6개월간격) |
| 피험자 동의 | ○ | 고주파  열치료 |  |  |  |
| 인구통계학적 정보 | ○ |  |  |  |  |
| 과거력 | ○ |  |  |  |  |
| 신체검진 | ○ |  |  |  |  |
| 활력징후, 체중, 신장^3)^ | ○ |  | ○ | ○ | ○ |
| Child-Pugh score | ○ |  |  |  |  |
| 진단검사^4)^ | ○ |  |  |  |  |
| 혈액검사 | ○ |  | ○^5)^ | ○^5)^ | ○^5)^ |
| U/A | ○ |  |  |  |  |
| Chest PA | ○ |  |  |  |  |
| 심전도 | ○ |  |  |  |  |
| F/U with CT |  |  | ○^6)^ | ○^6)^ | ○^6)^ |
| 합병증 확인 |  |  | ○ | ○ | ○ |
| 재발유무 확인 |  |  | ○ | ○ | ○ |
| 사망유무 확인 |  |  | **○** | ○ | ○ |

* 3개월 이후의 추적 검사는 고주파 열치료술 후 routine 추적검사에 준한다.

1) ‘피험자 동의’는 연구대상 선정 후 영상의학과 에서 이뤄진다. 그 외 시술 전에 이뤄지는 환자의 정보 및 검사는 내과와 외과등의 임상 의뢰과 에서 각각 이뤄진다.

2) 정해진 날짜에서 10일 전후로 한다.

3) 활력징후, 체중은 매 방문에서 평가하며 신장은 screening visit에서만 평가한다.

4) “대상 질환 및 대상자”의 임상적 진단기준에 따라 초음파, CT, MRI, 간동맥혈관조영술 중 택하여 시행한다.

5) CBC, LFT, AFP

6) 고주파 열치료를 시행한 경우 치료효과를 판정한다.

**동의서**

1. 본인은 본 임상시험 연구에 대한 시험목적, 예측효능, 시험방법, 이상반응, 시험참여에 따른 잠재적인 이점과 위험 등 시험에 관련된 제 정보들을 시험책임자(담당자)로부터 자세한 설명을 들었으며, 붙임 설명서를 통해 본 임상시험에 대해 충분히 이해하였습니다. 또한 본 임상시험은 연구 목적으로 수행된다는 사실을 알고 있습니다.
2. 본 임상시험을 지속적으로 참여하는데 영향을 줄 수 있는 새로운 정보가 수집되면, 시험책임자(담당자)는 관련된 모든 정보를 즉시 본인에게 알린다는 것을 알고 있습니다.
3. 본인이 임상시험의 세부사항에 대해 의문이 있을 경우, 언제라도 시험담당자에게 질문(문의)하여 충분한 답변을 받을 수 있음을 알고 있습니다.
4. 본인은 모니터요원, 점검을 실시하는 자, 심사위원회 및 식품의약품안전청장이 관련규정이 정하는 범위 안에서 임상시험의 실시 절차와 자료의 신뢰성을 검증하기 위해 본인의 의무기록을 직접 열람하는 데에 동의하며, 신분의 비밀이 보장되는 것을 알고 있습니다.
5. 본인의 임상시험 참여여부 결정은 자발적인 것이며, 임상시험 기간 중 언제라도 개인적인 사유 등으로 임상시험에의 지속적인 참여를 중도에 거부하거나 자유로이 임상시험 참가를 중단할 수 있으며, 이로 인해 본인이 원래 받을 수 있는 이익에 대해 어떠한 손실도 받지 않음을 알고 있습니다.

동의일 : 년 월 일

피험자성명 : (서명)

본인(시험자)은 위의 피험자에게 본 시험을 설명한 후 참가동의서를 받았음을 확인합니다.

서 명 일 : 년 월 일

시 험 자 : (서명)

**피험자 동의를 위한 설명서** (간세포암의 치료를 위한 내부냉각-식염수 주입 전극을 이용한 교대 단극성 고주파 열치료술)

귀하께서는 본 연구에 참여 하시도록 제안 받았습니다. 본 연구는 환자의 권리를 보호할 책임이 있는 서울대학교병원 의학연구윤리심의위원회의 승인을 받았습니다. 이 동의서는 왜 우리가 본 연구를 하며 귀하의 권리와 역할이 무엇인지에 대해 설명합니다.

귀하께서 본 연구에 참여하시기로 동의하기 전에 이 설명서를 읽고 이해하는 것이 중요합니다. 설명서에는 이 연구의 목적, 내용 및 방법, 예측되는 이점, 부작용, 그리고 주의사항 등이 포함되어 있습니다. 또한 귀하의 선택권과 참여중단에 대한 권리를 설명하고 있으며, 만일 귀하께서 본 연구에 참여 하신다면, 귀하는 이 설명서 및 동의서를 보관할 수 있도록 사본을 받게 되실 것입니다.

1. **임상시험 제목**

간세포암의 치료를 위한 내부냉각-식염수 주입 전극을 이용한 교대 단극성 고주파 열치료술: 예비 연구

1. **임상시험의 배경 및 목적**

원발성 간암은 위암, 폐암에 이어 국내 암 등록순위 3위인 암으로 특히 40, 50대 남자의 중요한 사망원인을 차지하며. 이중 간세포암 (Hepatocellular carcinoma)은 우리나라의 경우 원발성 간암의 약 90%를 차지합니다. 환자의 대부분이 자각증상이 생긴 후에 종양이 발견되고 대부분이 진단 당시에 간경변증을 동반하고 있어 예후가 불량하며. 또한 근치적 치료 후 재발이 잘 되어 계속적인 관리가 중요합니다. 최근 간세포암의 고위험군에 대한 선별검사가 과거에 비해 증가하여 소간세포암 (small HCC)의 비율이 증가하였으며, 치료방법들도 발달하여 치료성적이 향상되었습니다. 또한, 근래 국내 대장암의 발생빈도가 급격히 증가하고 있으며, 이와 함께 간으로 전이도 발생이 증가하고 있습니다.

간의 악성종양에 대한 치료법의 선택에 따른 예후는 논란이 많으나 최근 고주파 열치료술은 수술적 치료가 어려운 일차성 간암 및 이차성 간암의 치료를 위해 비침습적 국소 치료법의 하나로 널리 이용되고 있으며, 근래에는 폐암이나 신장암등에도 그 이용범위가 확대되고 있습니다. 실제 국내에 많은 간세포암의 경우 근치술은 수술적 절제술로 알려져 있으나 종양의 범위나 크기, 잔여 간기능의 저하등으로 수술의 대상이 되는 환자는 제한적입니다. 따라서, 이러한 환자에 대한 비술적 치료법에 대한 연구가 최근 활발하게 진행되고 있으며, 그 중 고주파 열치료술은 약 500 kHz내외의 교류성 전류를 종양에 가하여 조직내 이온들의 떨림에 의한 마찰열을 발생시켜 조직에 응고성 괴사를 유발시키는 방법으로 간종양의 국소소작에 있어서 효과적인 방법으로 각광을 받고 있습니다. 하지만, 기존의 고주파 열치료술의 중요한 한계점으로 거론되고 있는 것은 단위 소작당 소작면적이 2-3.5cm정도로 제한적 이다는 것이며, 이로 인하여 이로 인하여 종양과 종양 주변에 10mm 두께의 안전연을 형성하기 위해서는 2cm이상의 종양을 치료 시 여러 번 전극의 위치를 변경시켜야 하나 소작 중 발생하는 미세기포로 인하여 정확한 위치의 변경이 매우 어려워 충분한 안전연을 확보하는 완전소작의 가능성이 줄게 됩니다.

   상기 기술한 바와 같이 현재 수입되어 사용되고 있는 단극성 고주파 전극은 생체에서 3cm이상의 직경을 가지는 응고괴사를 만들기 어렵다는 점과 하나의 전극에만 고주파 에너지를 전달 할 수 있다는 점이 큰 제한 점 입니다. 따라서, 이러한 제한점을 극복하기 위해서 다전극침(clustered electrode, separable clustered electrode)등을 이용한 교대고주파 열치료술이나 다극성 또는 이극성 고주파 열치료술이 시도 되고 있으며, 본원에서는 최근 까지 서로 분리가 가능한 세개의 전극침을 가진 옥토퍼스 전극을 이용하여 단극성 교대 고주파 열치료술을 시행하고 있으며, 3년 국소 재발율이 11~15%정도임을 확인 하였다. 하지만 이러한 국소 재발 수치는 현재 까지도 수술의 국소 재발율 (~3%)에 비하여서는 국소 재발율이 높은 편이다. 이러한 결과는 RFA시술 중 전극 주변으로 전류의 집중으로 인한 숯형성이 발생하여 하나의 전극에 전달할 수 있는 최대 고주파 에너지가 점차 감소하기 때문으로 생각된다. 최근 이를 극복하기 위하여 시술 중 전극의 내부냉각과 함께 전극침의 미세 구멍으로 소량의 생리 식염수를 주입할수 있는 내부냉각-식국내 기술로 개발되어 국내외 병원에서 쓰이고 있으며, 이전의 동물실험 보고 및 임상연구에 의하면 생리식염수주입 전극을 이용한 다전극 고주파 열치료술은 기존의 단극성 고주파 열치료술에 비하여 단위시간당 큰 응고괴사를 만들 수 있으며, 이로 인하여 시술 시간을 단축할 수 있음이 기술되었다. 따라서, 이 전극과 교대 단극성 고주파 전달 방식를 이용하면 시간당 더 큰 부피의 괴사병변을 종양을 포함하여 만들 수 있으리라 예상되며, 단위시간당 고주파 에너지의 열전달 효율이 향상되어 시술시간이 짧아지고 더 큰 종양 주변 안전연을 안정적으로 형성하여 국소 재발율을 줄일 수 있을 것으로 기대된다. 이에 본 연구자는 최근 개발된 단극성 고주파 발생기와 생리식염수주입 전극을 이용한 교대 단극성 고주파 열치료술의 기술적 성공율, 12개월 국소 재발율과 평균 시술시간을 기존에 쓰이던 교대 단극성 고주파 열치술과 비교 평가하고자 합니다.

**내용 및 방법**

간세포암으로 새로이 진단받은 환자들을 대상으로 내과나 외과를 통해 영상의학과 에 고주파 열치료술을 받도록 의뢰된 환자 중 고주파 열치료술을 시행하는 영상의학과 전문의에 의해 본 연구에 적합한 환자인지 판명한 후, 선정된 환자분이 본 연구에 참여하기로 동의할 경우 이후 환자분은 단극성 다채널 고주파 발생기와 내부냉각관류전극을 이용한 교대단극성고주파 열치료술 또는 기존의 교대 단극성 고주파 열치료술 중 하나로 무작위 배정되어 배정된 방법에 따라 치료받게 됩니다.

1. **시술방법**

먼저 고주파 열치료술은 “모든 살아있는 세포는 섭시 50-60도이상의 온도로 1분이상 노출되면 단백질의 변성에 의해 죽는다”는 것에 의거한 열치료법의 하나입니다. 종양을 고온으로 죽이기 위해 체내의 종양 내에 바늘 형태의 전극을 위치시키고, 전극을 통해 +극과 –극이 매우 빠른 속도로 바뀌는 고주파를 체내에 전달 시키면 종양내의 +이온과 –이온들이 고주파전류를 따라 이동하는 떨림현상이 발생되어 섭시 60-100도의 마찰열이 유도됩니다. 중재적 초음파 시술이 가능한 초음파 실에서 영상의학과 전문의 2인이 시행하게 되며, 고주파 열치료를 하기 전에 얻은 CT나 MRI 영상에서 간세포암으로 진단되었던 병변을 초음파로 확인하고, 고주파 전극을 찌를 자리를 정한 후 소독과 국소마취를 합니다. 이후 초음파를 보면서 전극이 부착된 바늘을 미리 마취한 자리를 통해 삽입한 후 고주파를 발생시켜 10-40분 동안 종양을 태워 제거하게 됩니다.시술은. 시술 소용시간은 종양의 크기, 개수, 위치, 환자의 협조상태에 따라 다릅니다만, 기본적으로 2-5cm 종양의 경우에는 약 12-30분간 1-2회 시술로 종양을 치료할 수 있습니다. 시술 직 후 CT영상을 얻어서 간세포암 병변이 잘 치료가 되었는지 와 시술에 관련되어 합병증이 발생하였는지를 파악하고, 만약 잔존 병변이 있다면 이에 대해 바로 재시술을 시행하게 됩니다.

1. **시술 전의 주의사항**
   1. 본 연구에 참여하실 환자분들에게는 해당하지 않지만 일반적인 주의사항은 다음과 같습니다. 고주파 열 치료를 할 수 없는 환자는 심장, 폐, 간 등의 장기에 심한 기능장애가 있는 환자, 출혈성 경향이나 심한 복수가 있는 환자, 간 이외 장기에 전이가 있거나 간문맥에 종양혈전이 있는 환자, 간성 혼수 환자, 급성 감염이 있는 환자, 면역 저하 환자, 임산부 등이 있습니다. 대부분 시술을 받는 환자는 간경변증을 동반하고 있기 때문에 출혈성 경향이나 복수가 있는 경우가 많습니다. 이 시술은 비교적 굵은 바늘이 간을 뚫기 때문에 이런 환자에서 많은 출혈이 있을 수 있으므로 수술 전에 여러 검사를 통해 이상이 확인되면 이를 엄격하게 교정한 후 시술하게 됩니다.
   2. 검사 전날 밤 12시부터 금식을 시작하여, 시술 전까지 물도 마시지 말아야 합니다.
   3. 이 시술은 비보험이며 비교적 고가의 시술입니다.
2. **시술 후의 주의사항**
3. 시술 후 출혈을 방지하기 위해 시술한 부위를 모래주머니로 압박한 상태로 4시간 이상 절대 안정을 취해야 합니다. 경우에 따라서는 담당의사의 지시를 따르면 됩니다.
4. 시술 후 1개월째 혈액검사와 남아있는 종양이 있는지 확인하기 위해 CT 검사를 합니다. 이때 남아있는 종양이 있으면 재 시술을 하게 됩니다.
5. 이후에는 매 3개월마다 혈액검사와 CT를 2년 동안 시행합니다. 2년 이후에는 3개월마다 혈액검사를 시행하며, 방사선학적 검사는 초음파와 CT를 교대로 시행하여 최종시점까지 추적 관찰하게 됩니다. 추적관찰 기간 중 재발한 경우에는 이차적 연구종료에 해당하며 이 경우 의료진의 토의 하에 환자 분의 상태에 가장 적합할 것으로 판단되는 치료법을 제안할 것입니다. 상기한 치료법(수술적 절제술, 고주파 열치료)을 다시 시행할 수도 있고, 그 외에 경피적 에탄올 주입술, 경동맥 화학 색전술, 정맥용 항암제 투여 등을 시행할 수도 있습니다.
6. **시술 후 후유증 내지 합병증**
7. 시술 도중 통증을 호소하는 경우가 비교적 흔하게 나타나나, 그 정도는 개인마다 다양합니다. 이를 예방하기 위해 시술 전이나 시술 도중에 진통제를 투여합니다.
8. 이 시술은 비교적 안전한 시술로 인정을 받고 있지만 굵은 바늘을 간에 찌르고 고주파의 전류를 흐르게 하므로 출혈, 감염(복막염, 간농양), 천공, 화상 등을 일으킬 수 있고, 매우 드물지만 돌이킬 수 없는 육체적 사고 및 사망 등의 합병증이 발생할 수 있습니다. 이러한 시술은 기존의 고주파 열치료기를 이용한 고주파 열치료술에 동일하게 발생하수 있는 합병증입니다. 기존의 보고 및 본원의 경험에 의하면 입원기간의 연장을 요하거나 추가적인 중재적 시술를 요하는 합병증의 발생빈도는 2% 이하입니다. 사망률은 0.3%로 보고되었으며, 본원에서 400례의 경험으로는 사망한 예는 없었습니다.
9. **고주파 열치료 이외의 치료방법, 치료하지 않는 경우**
   1. 경동맥 화학색전술: 간동맥의 혈류를 차단하여 암종의 괴사를 유발합니다. 시행후 우상복부 동통과 발열을 초래하고 간의 효소치가 상승할 수 있습니다. 경동맥 화학 색전술의 경우 주요 적응증은 수술적 치료가 어려운 환자로서 간내에 여러 개의 종양이 있는 경우가 가장 대표적인 경우입니다. 간내에 단일종괴로 출현하는 간세포암의 경우에도 경동맥 화학 색전술을 시행할 수 있으나 많은 경우 잔존종양이 남을 수 있습니다. 기존의 보고에 따르면 단일 종괴에 대한 색전술 치료후 완전 괴사는 50%-60% 내외로 얻을 수 있는 것으로 보고되어있습니다. 고주파 열치료 술에 비교하여 장점은 여러 개의 종양을 동시에 치료할 수 있는다는 점이 있고, 단점으로는 1회치료로 완전 괴사를 만들 수 있는 가능성이 고주파 열치료술에 비하여 낮으며 종양의 완전괴사를 위해서는 흔히는 여러 차례의 시술을 필요로 합니다. 이에 반하여 고주파 열치료술은 대부분 1회 치료로 90%내외에서 완전괴사를 얻을 수 있는 치료입니다.
   2. 경피적 에탄올 주입술: 직접 괴사물질을 암종에 주입하여 괴사시키는 목적으로 무수 알코올을 초음파를 이용하여 주사하는데 조기 암종으로 크기가 2cm 이하로 작을 때 시행할 수 있습니다. 또한 고주파 열치료술과의 차이점은 2cm 내외의 종양이라 할 지 라도, 많은 경우 여러 번의 주입이 필요한 점(평균 2-3회) 입니다.
   3. 수술적 절제술: 간종양의 분포 및 잔존 간 기능등을 평가하여야 하며 수술적 절제술이 적응이 되면 현재까지 알려진 치료법 중 가장 치료성적이 좋습니다.
   4. 이외에도 화학 요법, 방사선 요법, 면역 요법, 상기 치료 방법들의 병합 요법 등 여러 시도가 있을 수 있습니다.

**연구 참여 기간**

고주파 열치료를 시행 받은 후 12개월 간 추적 관찰을 받게 됩니다.

**투여 약물**

고주파 열치료를 받는 동안 처치와 관련하여 투여되는 것 외에 본

연구를 위하여 특별히 투여하는 임상 시험용 의약품은 없습니다.

**환자의 경제적 부담**

본 연구는 지금까지 영상의학과 에서 시행하는 고주파 열 치료법과 시술 방법에 있어 거의 동일하고, 추적 관찰하는 동안 검사하는 방식도 동일합니다. 따라서 환자분이 본 연구 참여로 인하여 추가로 부담하는 것은 없으며, 오히려 고주파 열치료 시 사용되는 전극비용은 최소한 같습니다. 그리고 본 연구에 참여하더라도 연구 참여에 대한 경제적 보상은 없습니다.

**치료에 따른 합병증**

시술은 합병증을 동반할 수 있습니다. 합병증은 본 연구의 참여여부와 상관없이 고주파 열 치료술 시행 과정에서 발생할 수 있는 사항입니다. 일어날 수 있는 합병증으로는 출혈, 감염(복막염, 간농양), 천공, 화상 등을 일으킬 수 있고, 매우 드물지만 돌이킬 수 없는 육체적 사고 및 사망 등의 합병증이 발생할 수 있습니다. 본원에서의 경험으로는 추가적인 영상의학과 적 시술이나 수술적 치료를 요하는 합병증의 발생 빈도는 2%이하이며, 사망의 예는 없습니다. 만약 합병증이 발생할 경우 의료진은 최선을 다해 치료하여 환자분이 회복할 수 있도록 도와드릴 것입니다. 하지만 이에 따른 경제적 보상은 없습니다.

**예측되는 이점**

본 연구에 참여하시면 현재 본원에서 사용되고 있는 옥토퍼스 전극을 이용한 교대단극성 고주파열치료술 또는 생리 식염수 주입이가능한 내부냉각-식염수주입 전극을 이용한 교대 단극성고주파 열치료술을 받게 되며, 이 두 방법 모두는 타병원에서 주로 이용하는 단일 전극을 이용한 단극성 고주파 열치료술에 비하여 시술 시간이 짧고 단위 시간당 응고괴사면적이 큰 이론적 장점이 있습니다. 또한 만약 내부냉각관류 전극을 이용한 교대 단극성 고주파열치료술은 기존의 방법에 비하여 단위 시간당 응고괴사면적이 더 큰 이론적 장점이 있을 수 있습니다. 환자분의 모든 치료 과정과 치료 후 추적관찰을 받는 동안 담당 연구진에서 환자분의 외래 진료뿐만 아니라 추가적인 치료가 필요할 경우 등 모든 과정에 있어서 관리를 해드리기 때문에 보다 편리하게 진료를 받으실 수 있으며, 환자분의 상태에 대해서 궁금하신 사항이 있을 경우 담당 연구진으로부터 언제든지 설명을 들을 수 있습니다.

**자발적 참여 및 연구자에 의한 연구참여 중지에 대한 고지**

본 연구는 완전히 환자분의 자유 의사에 의한 것입니다. 따라서, 환자분은 본 연구에 참여하지 않을 수도 있고, 또한 언제라도 동의를 철회하고 참여를 중단할 수도 있으며, 그에 따른 어떠한 불이익이나 차별도 없을 것입니다. 환자분이 본 양식에 서명하거나 본 연구 참여에 동의함으로써 환자분의 법적 권리를 포기하는 것이 아니라는 사실을 다시 한번 알려드립니다. 또한 연구 도중에 피험자의 연구참여가 피험자의 동의가 없이도 연구자에 의해 중지되는 경우 및 해당사유, 피험자의 지속적인 참여의지에 영향을 줄 수 있는 새로운 정보가 수집되면, 즉시 피험자 또는 대리인에게 알려드릴 것입니다.

**피해발생시 보상 및 치료대책**

서울대학교병원은 본 연구 계획서에 따른 절차와 직접 관련되어 상해가 발생한 경우, 이에 대한 적절한 의학적 조치를 취할 것입니다. 그러나 그에 따른 경제적 보상은 없습니다. 단 이 연구에 사용되는 고주파 열치료기기와 직접적으로 연관되어 발생하는 피해는 피해자 보상 규약에 의거하여 연구자의 책임하에 보상될 예정입니다.

**자료보호**

본 연구의 모든 결과는 서울대학교병원의 순수 연구 목적과 환자 본인에게 통보해 드리기 위한 목적으로만 기록되며, 모든 자료는 엄격하게 환자의 비밀이 유지되어 보호를 받게 됩니다. 환자분이 본 연구에 참가하기로 동의할 경우, 본 연구에서 수집된 자료는 익명으로 다뤄지며 관계 기관에 제출될 수도 있습니다.

언제라도 본 연구에 대한 의문사항이 있을 때에는 담당의사 (연구책임자) 이 정민이나 영상의학교실(문의전화: 02-2072-0694)로 알려주시기 바랍니다. 또한 만일 환자분이 본 연구에 참여하는데 대하여 피험자의 권리에 관해 의문이 있으면 서울대학교병원 의학연구윤리심의위원회(문의전화: 02-2072-1601)로 문의하시기 바랍니다.

☞ 임상시험책임자 : 성 명: 이정민

연구간호사 : 이현희, 연락처: (02) 2720- 2519

☞ *** 연구비 내역서

|  | | 금액 (천원) | 구성비 (%) | 비고 |
| --- | --- | --- | --- | --- |
| 1. 인건비  연구 간호사 (1인) | | 6,000  1000 X 6개월 | 20% | 동의서 취득  자료 관리 및 입력  CT 및 RF parameter 입력, 자료분석 |
| 2. 자문료 | 영어교정  통계자문료  IRB 심사료 | 2,000  500  500 | 10% | 향후 결과 분석시 통계자문 및 논문 작성시 영어 자문 |
| 3. 재료비 | 동영상 편집 프로그램 또는 렌즈 | 1,500 | 10% | RF parameter의 real time monitoring 및 자료 관리  RF planning 및 fusion postprocessing |
|  | 이동식 하드디스크 및 USB저장매체 | 500 |  |  |
|  | 이동식 Tablet PC (동의서 설명 보조) | 1,000 |  |  |
| 4. 기술 정보 활동비  회의비  도서구입비  자료 구입비, 자료 복사비 | | 6,000 | 20% |  |
| 5. 여비  구미학회 항공료 및 체재비  주 연구자: 1회  공동연구자: 1회 | | 6,900 | 23% |  |
| 6. 수용비 및 수수료 | | 1,500 | 5% |  |
| 7. 직접비 총합계 (88%) | | 26,400 | 88% |  |
| 8. 간접비 (12%) | | 3,600 | 12% |  |
| 9. 총 연구비 (100%) | | 30,000 | 100% |  |
| 10. 병원관리비 (5%) | | 6000 | 5% |  |
| 11. 전체 연구비 (105%) | | 30,600 | 105% |  |
