# Supplementary material for: Comparison of switching bipolar ablation with multiple cooled wet electrodes and switching monopolar ablation with separable clustered electrode in treatment of small hepatocellular carcinoma: A randomized controlled trial
Source: PLoS One. 2018 Feb 8;13(2):e0192173. doi: 10.1371/journal.pone.0192173 (PMC5805261; doi:10.1371/journal.pone.0192173)
Supplement: S3 File — (DOCX) [file pone.0192173.s003.docx]

**Switching Bipolar Radiofrequency Ablation Using Cooled-Wet Electrode for Treatment of Hepatocellular carcinoma: a Preliminary Study**

**Institution: Department of Radiology, Seoul National University Hospital**

**Principal Investigator: Professor Jeong Min Lee**

**1. Study Title:**

Switching Bipolar Radiofrequency Ablation Using Cooled-Wet Electrode for Treatment of Hepatocellular carcinoma: a Preliminary Study

**2. Purpose**

To determine safety, ablative zone, technical success rate and early safety data of recently introduced cool-wet electrode in eligible patients who are indicative for radiofrequency ablation (RFA) for liver tumors.

**3. Target disease and subjects**

**1) Study design**

Among the patients with less than 3 hepatocellular carcinomas, patients who were considering radiofrequency ablation(RFA) were divided into two groups. In one group, multichannel radiofrequency treatment using conventional octopus electrode (SM-RFA) and another group underwent radiofrequency ablation using cooled wet electrode and switching bipolar mode (SB-RFA). This is a randomized study to investigate the ablation volume, the technical success rate, the short-term recurrence rate, and the average procedure time per unit session in both treatments.

**2) Study population**

A total of 70 evaluable subjects who met the study inclusion criteria and submitted the informed consent to the study were enrolled. A total of 70 subjects were enrolled in the control group and the experimental group. According to previous reports, the treatment results of RFA have been reported to differ according to the size of the tumor. All 70 patients will be recruited considering the size of the tumor (1 ~ 2.5cm and> 2.5cm).

The purpose of this study was to investigate the difference in local recurrence rate after 12 months. But there is no basis for the local recurrence rate expected in each group, so we did not calculate the number of subjects for securing the accuracy of the power or estimation. Of the patients newly diagnosed with CT or MRI on HCC for a year, about 150 patients are considered for high-frequency thermal therapy. Based on this, the target number of subjects agreed with the study, and 70 patients (35 per each group) who satisfy both the selection criteria and the exclusion criteria are selected.

The total number of candidates should be 77 in consideration of the 10% dropout rate.

Subjects will be randomly assigned to a test group (Switching bipolar mode with three cooled wet electrodes) and a control group (switching monopolar mode with a separable clustered electrode). The study subjects will be assigned into the test group and the control group with a ratio of 1: 1. The randomization was performed by a third party unrelated to the study, using a blocked randomization method with a block size of 4. The size of the tumor (1 to 2.5 cm and> 2.5 cm) is also considered at randomization.

The random assignment is performed using a pre-written randomization table and administered and managed by the Medical Research Collaborating Center of Seoul National University / Seoul National University Hospital.

**Inclusion criteria**

1) Accept the protocol requirements and submit the agreement.

2) 20-75 years old

3) Patients with liver cirrhosis who underwent MDCT or MRI within 60 days of suspected hepatocellular carcinoma of 1 cm-5 cm size

4) The index tumor should not have been previously treated with other local therapies.

** Criteria for diagnosis of hepatocellular carcinoma

1) with risk factors (HBV positive, HCV positive, liver cirrhosis, etc.)

2) When one or more of the CT scans (dynamic CT) and dynamic MR imaging (dynamic MRI) show favorable findings for hepatocellular carcinoma

3) HCC is diagnosed by histologic examination

4) Primovist enhanced MRI showed contrast enhancement in the arterial phase and a contrast defect in the hepatocyte specific phase

**Exclusion criteria:**

1) When the number of malignant liver tumors is 3 or more

2) When the maximum tumor size is 5 cm or more (diffuse carcinoma)

3) When the tumor is attached to the central liver portal vein or hepatic vein more than 5mm

4) Severe hepatic failure (Child-Pugh grade III)

5) In case of invasion of liver vessel by malignant liver tumor

6) Severe coagulopathy (prolongation of 50% or more of INR for platelets below 80,000/㎣)

7) In the case of multiple extrahepatic metastases

8) A situation where the probability of obtaining appropriate data for the purpose of the study is very low

**4. Expected Study Duration**

1) IRB approval date ~ 2015.02.28: subject registration and RFA treatment

2) IRB approval date ~ 2018.02.28: follow-up of subjects and the final data analysis

**5. RFA treatment & Follow Up**

1. **RFA planning**

Multiphasic or perfusion CT and Primovist MRI were used to determine the location of the tumor. The US-CT-MR fusion systems of Esaote, Phillips, GE or Siemens will be used during RFA treatment, Through these fusion systems, the location of the electrodes to be installed on the tumor, the number of electrode insertion, and a safe access route are planned.

1. **RFA treatment**

In the control group, 200 W switching RF generator (Viva Multi generator: Star Med, Korea), Octopus electrode 15 gauge or 17 gauge (Star Med, Korea), and ground pad and pump for internal cooling. In the test group, RF ablation system(M3004 ,RF medical co.) and three 15-gauge or 17-gauge cooled-wet electrodes were used without internal grounding pad.

The procedure was the same in both groups. Two or three RFA electrodes were placed and cooled with saline solution and the tumor was ablated for about 10-30 minutes depending on tumor size. In the test group, saline will be injected through the two holes on the active surface of the electrode for 1cc / 10min.

In the Fusion US image, the electrodes are placed in the tumor through the planned path of the electrodes. The high-frequency energy is then transferred to one or two electrodes with switching bipolar or monopolar modes.

1. **Follow Up**

- CT is scheduled to be performed immediately after RFA. Pre-RFA CT / MR images and CT images after RFA are registered by Siemens Hepacare program for evaluation of complete necrosis of Tumor. We will assess whether total necrosis has occurred. If a residual mass is identified or insufficient safety margin is not available, additional procedures should be performed and assessed.

- AFP or CEA, CBC, LFT and CT are performed at 1 month after RFA.

- Blood tests and CT are performed at 3-to-6-month intervals for up to 3 years

**5. Evaluation**

1. **Primary Outcome Measures**

Local recurrence of tumor over the first 12 months after RFA

1. **Secondary Outcome Measures**

A. Maximum diameter and volume of nonenhancing ablation area on CT

B. Technical success one month after the procedure on CT

C. Average procedure time and incidence of complications

D. Distant recurrence rate and metastatic incidence at 12, 24, and 36 months after the procedure

E. Local recurrence rate at 24, and 36 months after the procedure

F. Evaluation of treatment success using registration tool

G. Evaluation of registration accuracy of US-CT-MR fusion images

H. Comparison of ablation volume per time according to electrode size: Comparison between 15 gauge electrode and 17 gauge electrode

**6. Safety**

Presence and severity of complications after RFA treatment is evaluated to use the Clavien system.

**7. Statistical Analysis**

**1) Primary Outcome Measures**

Local recurrence: Kaplan-Mayer survival analysis using the Mantel-Cox test or the Breslow test .

**2) Secondary Outcome Measures**

A) Maximum diameter and volume of nonenhancing ablation area on CT: independent T-test or Wilcoxon rank sum test

B) Technical success: chi-square or Fisher's exact test

C) Average procedure time: independent T-test or Wilcoxon rank sum test

D) Incidence of complications: chi-squire or Fisher's exact test.

E) Local recurrence rate, distant recurrence rate, metastases rate: Kaplan-Mayer survival analysis using the Mantel-Cox test or the Breslow test .

F) Evaluation of treatment success using CT registration tool: Calculated as a percentage

A) Comparison of ablation volume per time: Independent T-test or Wilcoxon rank sum test
